# Photoswitching behaviour of quinoline-substituted bicyclooctadienes and the role of protonation

**DOI:** 10.1039/d6cp00936k

**Published:** 2026-04-14

**Authors:** Paul A. Gueben, Jacob Lynge Elholm, Rebecca J. Salthouse, Pedro Ferreira, Thomas Robert, Pascal Gerbaux, Kasper Moth-Poulsen

**Affiliations:** a Department of Chemical Engineering, Universitat Politècnica de Catalunya, EEBE, Eduard Maristany 10–14 Barcelona 08019 Spain kasper.moth-poulsen@upc.edu; b Organic Synthesis and Mass Spectrometry Laboratory (S^2^MOs) Chemistry Department, University of Mons – UMONS, 23 Place du Parc B-7000 Mons Belgium Pascal.GERBAUX@umons.ac.be; c The Institute of Materials Science of Barcelona, ICMAB-CSIC, Bellaterra Barcelona 08193 Spain; d Department of Chemistry and Chemical Engineering, Chalmers University of Technology Gothenburg SE-41296 Sweden; e Catalan Institution for Research & Advanced Studies, ICREA, Pg. Lluis Companys 23 Barcelona 08010 Spain

## Abstract

Molecular Solar-Thermal (MOST) systems employ photoswitches that convert solar energy into chemical energy in the form of a metastable isomer and release it as heat upon triggering. An example of such a photoswitch is bicyclooctadiene (BOD), which is converted into its higher energy photoisomer, tetracyclooctane (TCO), by a [2+2] cycloaddition upon UV light irradiation. Despite their potential as MOST candidates due to a high calculated energy storage density of ≈ 1.77 MJ kg^−1^, BODs are underinvestigated due to their retro Diels–Alder triggered degradation upon heating and very short half-lives of the TCOs in the order of seconds to minutes. Here we report the synthesis of three new acceptor–acceptor BOD isomers substituted with quinoline and the characterisation of their photophysical and photochemical properties. Two of the three BODs exist as a pair of rotational conformers with different absorption profiles, shown experimentally and supported by computational analysis. Calculated storage energies are found to be slightly higher than those of comparable naphthalene-substituted BODs, ranging from 144.0 to 163.6 kJ mol^−1^. We demonstrate that the substitution position of the quinoline has an influence on key MOST-relevant optical properties including thermal half-lives which range from 13 s to 6 min and the UV-vis absorption spectra. Protonation of the quinoline moiety induces a red shift of ≈ 40 nm in the absorption spectra of each BOD and leads to divergent behaviour upon irradiation, leading to photoswitching, fluorescence, or degradation, depending on the position of the nitrogen in the quinoline ring. These experimental and computational results elucidate how quinoline substitution and nitrogen position govern structure–property relationships in BOD photoswitches, providing design principles for further tuning and improving BODs and other photoswitches towards MOST application.

## Abstract 2

Quinoline-substituted bicyclooctadienes for MOST application show protonation-tuned properties, inducing red-shifted absorptions and isomer-dependent photochemical responses including photoswitching, fluorescence and a light-triggered side reaction.

## Graphical abstract

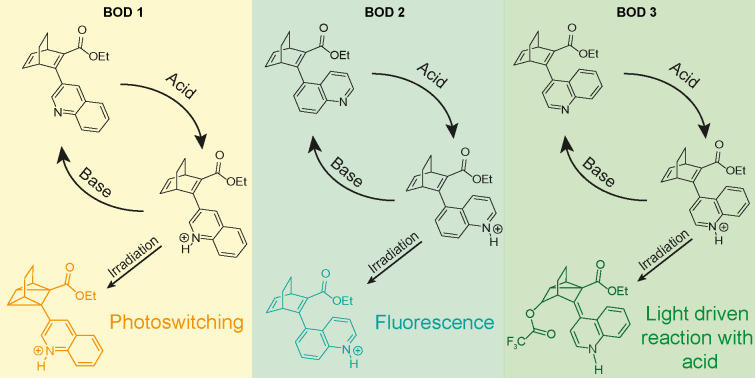

## Introduction

**Fig. 1:**
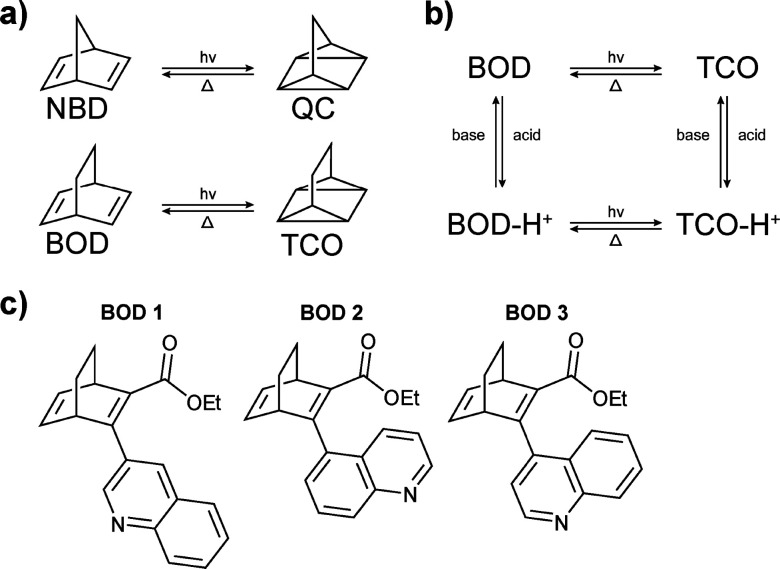
(a) The norbornadiene (NBD)/quadricyclane (QC) system and the bicyclooctadiene (BOD)/tetracyclooctane (TCO) system. (b) Diagram showing the equilibrium between BOD and TCO as well as their protonated forms; there are four possible states for each derivative. (c) BODs isomers synthesised for the current work.

Reducing the consumption of fossil fuels and the emission of greenhouse gases can be achieved by transitioning to more sustainable and renewable energy sources. In this context, investigating new ways of harvesting and storing solar energy is important. One strategy that combines these two features is the use of molecular solar thermal (MOST) energy storage systems.^1–3^ These systems are employing molecular photoswitches able to undergo photoisomerization to a meta-stable higher energy state, storing solar energy in the form of chemical energy, and releasing it as heat on demand,^4^ that can be triggered thermally, catalytically or electrochemically.^5,6^ In this framework, several photoswitches have been studied with azobenzene derivatives,^7–10^ the dihydroazulene/vinylheptafulvene system (DHA/VHF)^11–14^ and the norbornadiene/quadricyclane couple (NBD/QC)^15–19^ being considered as the most promising candidates. Despite these three frontrunners leading the field, new types of photoswitches are continuing to be developed.^20^ For example, recent works investigated anthracenes,^21^ azaborines^22^ or hydrazones^23^ as potential MOST candidates. Nevertheless, the NBD/QC system exhibits attractive properties for MOST applications, such as excellent photostability over many photoswitching cycles,^24,25^ a low molecular weight and high energy density (up to ≈ 1 MJ kg^−1^) owing to the strained structure of the QC photoisomer.^26^ However, recent computational works identified bicyclooctadiene (BOD), which can be isomerised to tetracyclooctane (TCO) upon irradiation, as a promising candidate in terms of solar capture efficiency among the bicyclic systems (Fig. 1a).^27–32^ Despite the similarities in the structure of NBD and BOD, *i.e.* the additional methylene group within the bridge unit, their similar photoconversion mechanism and the absorbance in the UV/visible range for the unsubstituted parent molecules, the storage energy has been predicted to be much higher in the BOD/TCO system (up to ≈ 1.77 MJ kg^−1^) compared to the NBD/QC couple (≈ 0.97 MJ kg^−1^).^28^ Nonetheless, the BOD/TCO system has been less explored for several reasons, including the generally short half-life of the TCO isomers, ranging from seconds to minutes, and the irreversible degradation of BOD molecules into aromatic compounds when subjected to typical Diels–Alder temperatures (around 100 °C).^32^ Quant *et al.* reported a synthesis protocol for BODs to reduce their degradation using lower temperatures,^32^ though further studies to design BODs with improved half-lives as well as red-shifted absorption to better overlap with the solar spectrum would prove invaluable. It has been shown that incorporating donor/acceptor groups or acceptor/acceptor groups across one of the double bonds of NBD can lead to a red-shift in the absorbance.^33,34^ Moreover, the addition of bulky substituents, for example *ortho*-substituted phenyl rings, on NBD can enhance the half-life of the QC photoisomer by sterically restricting side-group rotation in the QC form, increasing the rotational barrier and destabilising the back-conversion transition state, thereby raising the activation entropy and free energy barrier.^18^

Another bulky substituent which has been shown to enhance the thermal half-lives of QCs or TCOs is naphthalene.^18,32^ Building on this precedent, we propose quinoline as a promising alternative bulky substituent for BOD. Quinoline is an electron-withdrawing heteroaromatic system comparable to naphthalene but with the added functionality of a ring nitrogen that enables further modifications (*e.g.* protonation). Quinoline derivations have been widely exploited for their optical properties in various fields such as OLEDs, photovoltaics and biosensing.^35,36^ Here, we envision quinoline to serve as both an electronic acceptor to red-shift the absorbance of BOD, and as a bulky substituent to increase the half-life of the TCO photoisomer.

Moreover, we speculate that the presence and position of the quinoline nitrogen atom will introduce an additional handle for tuning the photophysical behaviour.^37^ Protonation of the heterocycle is expected to further modulate the electronic structure, and by analogy to pyridine-substituted NBDs, where protonation has been shown to effectively “lock” the system in the high-energy metastable state,^38^ this strategy may offer a route to stabilise otherwise short-lived TCOs. This possibility to reversibly pass from the protonated to non-protonated state could therefore provide more control over the switching behaviour through the addition of acid/base as stimuli as well as light, giving multi-mode switches (Fig. 1b). The objective of the present study is to expand the knowledge of the structure–property relationship in BODs, including the impact of protonation over the (back)switching properties while offering new synthetic MOST candidates. With these design strategies in mind, here we report the synthesis and photophysical analysis, both experimentally and computationally, of three quinoline-substituted acceptor–acceptor BOD switches (Fig. 1c) both in their neutral and protonated forms (Fig. 1b) to evaluate their potential as MOST candidates.

## Result and discussion

### Synthesis

**Scheme 1:**
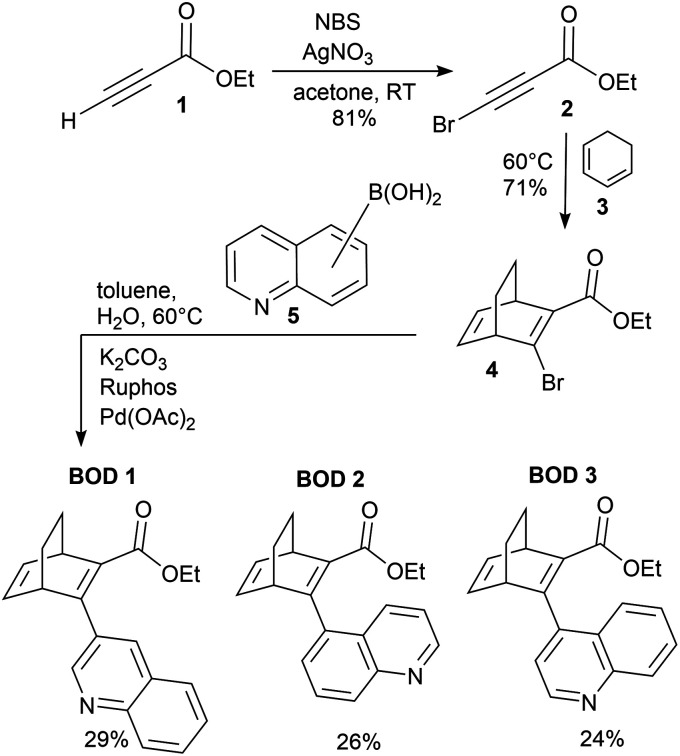
Reaction pathway for the synthesis of BOD 1, BOD 2 and BOD 3.

**Fig. 2:**
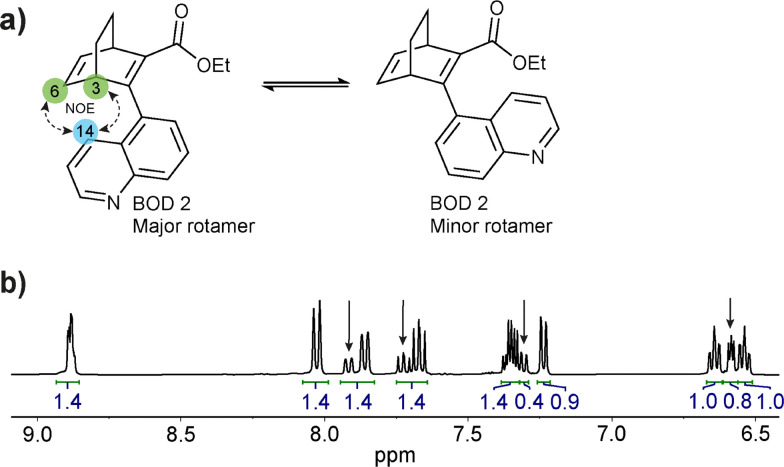
(a) Major and minor rotamers found for BOD 2. (b) Zoom on the aromatic part of the ^1^H-NMR spectrum of BOD 2 in CD_2_Cl_2_ in which signals from the minor rotamer are visible (arrows), and the integrals used to determine the ratio between the major and the minor rotamers.

The synthesis of the three studied compounds was inspired by Quant *et al.* (Scheme 1).^32^ They reported a single-step synthesis of BODs involving a Diels–Alder reaction between 1,2-cyclohexadiene and an electron-poor alkyne. Following the same protocol, as shown in Scheme 1, we synthesised compound 4 through a Diels–Alder reaction between cyclohexadiene and a brominated alkyne. Compound 4 was then used in various Suzuki coupling reactions with commercial boronic acids to substitute the bromine atom with different quinolines (Scheme 1). Both the Diels–Alder and Suzuki coupling reactions were carried out at 60 °C since higher temperatures can trigger retro Diels–Alder reactions leading to irreversible degradation of the BOD into an aromatic product plus ethene.^32^BOD 1, BOD 2 and BOD 3 were obtained from the Suzuki coupling in moderate yields (24% to 29%). More information relative to the synthesis is available in SI. Interestingly, BOD 2 and BOD 3 were both isolated as a pair of rotational conformers (rotamers). The ratio between the two rotamers was determined from NMR as 1 : 2.5 for both compounds (Fig. 2b). Similar ratios were determined by Quant *et al.* for the analogous naphthalene-substituted BOD, which indicates that the presence and/or position of the nitrogen atom in the ring does not affect the relative rotamer proportion.^27^ This behaviour was confirmed by variable temperature NMR studies starting from room temperature to 135 °C. Indeed, we observed the rotamer signals are reversibly merging with increasing/decreasing temperature (Fig. S2.13–S2.16). This suggests the presence of a thermal equilibrium between both rotamers. Interestingly, during the same experiment, the retro Diels–Alder byproduct started to appear in the NMR spectrum when the temperature reached 120 °C and its signals remained unchanged after the cooling showing the irreversibility of this degradation pathway (Fig. S2.17 and S2.18). The structures of the major and minor rotamer were assigned using nuclear Overhauser effect spectroscopy (NOESY) revealing correlation signals between H-14 and H-6 and H-3 (Fig. 2 and Fig. S2.11, S2.12).

Furthermore, the energy barrier between the rotamers was also determined, both experimentally and computationally. The barrier between the rotamers can be calculated with the data from variable temperature NMR experiments. The exchange rate calculated from the initial chemical shifts of both species and the temperature at which their signals coalesce can be introduced in an Eyring equation to obtain the thermodynamics of the energy barrier.^39^ With this experimental method, the energy barrier between the major and minor rotamer (Δ*G*^‡^_rota_) of BOD 2 and BOD 3 were found to be 76.1 kJ mol^−1^ and 71.8 kJ mol^−1^, respectively (Table 1 and Fig. S2.19). Computationally, a scan was made over the dihedral angle of the quinoline moiety, and two peaks were located along the 360° rotation (Fig. S5.1). The structures with the highest energy of each peak were assigned to the transition states. The rotamer energy barrier was determined as the energy difference between the ground state isomer of the BOD (major rotamer) and the transition state to the minor rotamer. Two pathways were identified for this rotamer (outwards and inwards), and the barrier was determined as the pathway with the lower energy cost. The values were found to be 80.3 kJ mol^−1^ and 78.3 kJ mol^−1^ for BOD 2 and BOD 3, respectively, in good agreement with the experimental values. The computational technique also allowed the determination of the rotational barrier for BOD 1 (25.8 kJ mol^−1^), which is significantly lower than those of BOD 2 and BOD 3. The calculated storage energy, Δ*G*_storage_, of each BOD (TCO to BOD conversion energy) is also reported in Table 1, and these values were determined computationally as the energy difference between the corresponding BOD and TCO. The values are similar for the three BODs with BOD 1 storing the highest amount of energy with 163.6 kJ mol^−1^ followed by BOD 3 with 153.9 kJ mol^−1^ and lastly BOD 2 with 144.0 kJ mol^−1^. These values are slightly higher than those of the naphthalene-substituted counterparts (152.9 kJ mol^−1^ and 142.5 kJ mol^−1^) which were also obtained through calculations.^32^ Unfortunately, the very short half-life of the TCOs and the high temperature sensitivity of the BODs prevent an experimental value for the storage energies from being obtained.

**Table 1:** Energy barrier between the minor and major rotamers collected experimentally and computationally and calculated storage energy for each BOD

BOD	Δ*G*^‡^_rota_ (kJ mol^−1^)		Δ*G*_storage_ (kJ mol^−1^)
	Experimental	Calculated	Calculated
1	—	25.8	163.6
2	76.1	80.3	144.0
3	71.8	78.3	153.9

### Photophysical properties

#### UV-vis absorption

**Fig. 3:**
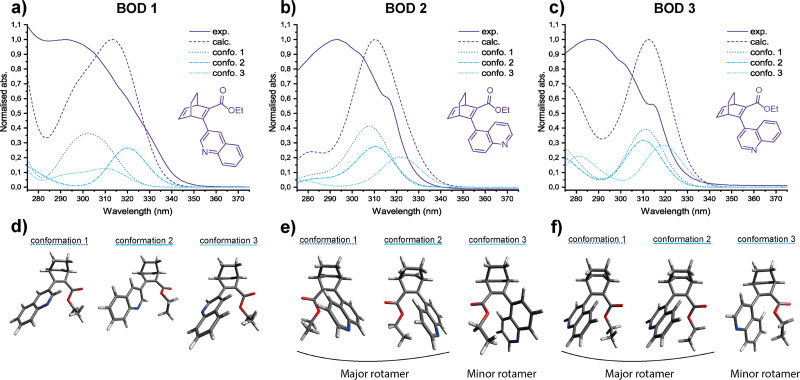
Comparison between the experimental and calculated weighted UV-vis absorption spectra for (a) BOD 1, (b) BOD 2, (c) BOD 3 and the 3D conformations of the three most stable conformers for (d) BOD 1, (e) BOD 2, (f) BOD 3 (larger figures available in SI).

The UV-vis spectra of the three BODs were recorded in acetonitrile at room temperature, as shown in Fig. 3. The calculated spectra of the three most stable conformers for each BOD derivative are also presented in the figure and were simulated using the method described in the theoretical section of the SI. The dashed curves correspond, for each BOD, to the calculated UV-vis spectra merging the weighted contributions (Fig. S5.2–S5.4) for each conformer considering their relative proportions (Fig. 3 and Table 2). For BOD 1, a broad peak is observed experimentally with a maximum at 292 nm and an onset at 351 nm. Meanwhile, BOD 2 shows an absorbance spectrum with a maximum at 293 nm and a shoulder at 316 nm, with an onset at 336 nm. A similar experimental spectrum has been recorded for BOD 3 with a maximum at 287 nm, and a shoulder at 315 nm with an onset at 346 nm. Generally, the three BODs absorb in the UV with BOD 1 being the most red-shifted and BOD 2 the least. The molar extinction coefficients were also determined (Table 2), and BOD 1 has the highest value, followed by BOD 2 and BOD 3. The maxima in the calculated spectra of the three BODs are all red-shifted by ≈20 nm compared to the experimental data and their shape follows the weighted contribution of the different conformers. For BOD 1, the 3 most stable conformers are distinguished by 3 different rotational orientations of the quinoline moiety (Fig. 3d and Fig. S5.2). Their structural similarity leads to strongly overlapping spectral features, such that the experimentally observed UV-vis spectrum appears as a broad peak. The cases of BOD 2 and BOD 3 are particularly interesting as the rotamers identified by NMR can also be distinguished by UV-vis spectroscopy. Initially, it was hypothesised that the shoulder in each spectrum corresponds to the minor rotamer, which is confirmed by calculations. In both systems, conformers 1 and 2 share the same rotational orientation of the quinoline moiety relative to the BOD core, differing only in the orientation of the ester methyl group. As a result, these two conformers display very similar calculated absorption profiles and together constitute the major rotamer. Conversely, conformer 3 shows a distinct rotational orientation of the quinoline moiety which shifts the absorption spectrum towards the red and gives rise to the experimentally observed shoulder (Fig. 3e, f and Fig. S5.3, S5.4). In summary, for BOD 2 and BOD 3, conformers 1 and 2 together compose the major rotamers, whilst conformer 3 corresponds to the minor rotamer.

**Table 2:** Experimental and calculated absorption profiles in acetonitrile. Absorption onset (*λ*_onset_), absorption maxima (*λ*_max_) and molar extinction coefficient at the absorption maxima (*ε*_max_) for BOD 1, BOD 2 and BOD 3

BOD	Experimental			Calculation
	*λ*_max_ [nm]	*ε*_max_ [M^−1^ cm^−1^]^c^	*λ*_onset_ [nm]^d^	*λ*_max_ [nm]
1	292	8230	351	313
2	293	6650	336	310
	316^a^	4020	—	321
3	287	5800	346	312
	315^b^	3130	—	319

a *λ*_max_ at the shoulder in the UV-Vis spectrum of BOD 2 assigned to the minor rotamer.

b *λ*_max_ at the shoulder in the UV-Vis spectrum of BOD 3 assigned to the minor rotamer.

c Calculated as the slope of Abs. *vs*. concentration for five different concentrations (Fig. S4.1).

d Determined as log(*ε*) = 2.

#### Photoisomerization

**Fig. 4:**
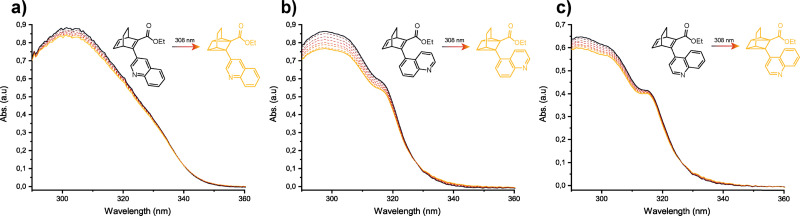
UV-vis absorption spectra for the irradiation of (a) BOD 1, (b) BOD 2 and (c) BOD 3 at 0 °C in acetonitrile (≈ 10^−4^ M), with a 308 nm LED.

Solutions of the BODs were prepared in acetonitrile (≈10^−4^ M) and subjected to irradiation with a 308 nm LED while the temperature was maintained at 0 °C to minimise the back conversion owing to the short thermal half-lives (Fig. 4). A gradual decrease in the absorption is observed for all BODs as well as an even weaker increase in absorption around ≈350 nm for BOD 1 and ≈340 nm for BOD 2 and BOD 3 (zoomed figures available in SI, Fig. S4.12). We propose that these bands belong to the different TCOs as supported by their calculated absorption spectra (Fig. S5.8). Isosbestic points are observed around 340 nm for BOD 1 and 325 nm for BOD 2 and BOD 3. This attests to the existence of a preferential photoproduct and to a clean conversion without degradation. The very subtle spectral changes observed even at low temperature could be explained by competitive absorption between BOD and TCO. To investigate this, we calculated the absorption spectra of the TCOs (Fig. S5.8); there exists an overlap between the calculated spectra of the TCOs and the experimental spectra of the BODs, in particular for BOD 2 and BOD 3. While the same overlap is not clearly visible for BOD 1, we speculate that it could still exist, as experimental bands are usually broader than those predicted by calculations. Additionally, for BOD 2 and BOD 3, it seems that the magnitude of the gradual decrease in absorption differs between the bands associated with the major/minor rotamers, indicating that the conformers could have different quantum yields. However, this effect cannot be quantified owing to the low resolution due to overlapping signals.

#### Quantum yield

The photoconversion quantum yields of the three BODs were obtained using an in-house automated set-up^40^ in the low concentration regime. The compounds were irradiated at 308 nm in acetonitrile at 5 °C, though for BOD 1 the irradiation was carried out at 0 °C to avoid spectral noise. The decrease in absorbance was monitored until the photostationary state (PSS) between BOD and TCO was reached *i.e.* when no further decrease was observed (note that, due to the short half-life, the degree of conversion at this equilibrium is dependent upon several factors, including concentration and photon flux). The details regarding the experiments are provided in the SI (Fig. S4.8). The quantum yields of each BOD are reported in Table 3. The quantum yields range from 2% to 5%, which are lower than the value of the structurally similar previously reported 1-naphthalenyl derivative that reaches 19% in toluene.^32^ Computational methods could help to predict molecular candidates that exhibit higher quantum yields, however the multireference character of the excited state of these systems makes calculations more challenging and beyond the scope of this study.^41^

**Table 3:** Photoconversion quantum yields in acetonitrile of the three BODs and half-lives of the three TCOs in acetonitrile and toluene at 25 °C

BOD	Quantum yield (%)	TCO	Half-life, *t*_1/2_ (s)	
			Acetonitrile	Toluene
1	5.1 ± 0.2	1	12.8	3.9
2	2.9 ± 0.2	2	382.8	171.0
3	2.2 ± 0.2	3	144.6	121.8

#### Back conversion

The rates of the thermal back-conversions from TCOs to BODs were measured at four different temperatures and the thermodynamic parameters were extrapolated from Arrhenius and Eyring analyses (Fig. S4.2–S4.4). As shown in Table 3, the half-lives of the TCOs span from a few seconds to several minutes. The experiments were carried out in two different solvents, namely acetonitrile and toluene, to assess any potential solvent effect on the half-life. As expected, BOD 2 and BOD 3 have a longer half-life than BOD 1 in both solvents. For both BOD 2 and BOD 3, the quinoline moiety is closer to the BOD core, hindering the back-conversion to a greater extent compared to BOD 1, for which the quinoline moiety sits further away from the BOD core. This behaviour corroborates the properties observed for naphthalene-substituted BOD/TCO and NBD/QC systems.^18,32^ Generally, the half-lives of all TCOs are shorter in toluene than acetonitrile with the most noticeable difference observed for BOD 2, whereas BOD 3 shows a similar half-life in both solvents. This indicates that the position of the nitrogen in the quinoline ring and the choice of solvent affect the half-life of TCOs.^42^ This behaviour could arise from a change of polarity with the position of the nitrogen atom giving a different interaction with the media and ultimately affecting the back reaction and the half-life, though further studies with more solvents would be necessary to draw conclusions.

#### Cyclability

Three BOD solutions in acetonitrile were irradiated with a 308 nm LED to form the corresponding TCOs, then let to thermally switch back to the BODs as followed using UV-vis spectroscopy. 7–10 cycles were performed for each of the BODs. For BOD 1, the whole experiment was carried out at 0 °C while the temperature was kept at 5 °C for the switching of BOD 2 and BOD 3 then increased to 40 °C to activate the back reaction. The temperature was then brought back to 5 °C to complete the cycle since a temperature effect was observed where the absorbance would decrease during the heating from 5 °C to 40 °C (Fig. S4.7). Overall, the three BODs were observed to be quite stable throughout the cycles with little to no degradation (Fig. S4.6).

## Effect of protonation

Probing the property changes induced by protonation was a key motivation for selecting quinoline as a substituent as it offers the possibility to explore external control of the switching through the addition of acid or base. Protonation of the quinoline moiety was carried out by adding 0.5 to 20 equivalents of trifluoroacetic acid (TFA) to solutions of the three BODs in acetonitrile. The resulting changes upon protonation were assessed by UV-vis and NMR spectroscopy, and the photoswitching behaviour of the protonated BODs was subsequently investigated.

### NMR spectroscopy

The NMR spectra of the three BODs were recorded in acetonitrile and titrated with several equivalents of acid to show the impact of the protonation on the NMR signals (Fig. S2.20 and S2.21). The most impacted signals correspond to the protons of the quinoline moiety which shift downfield upon addition of acid, suggesting that the nitrogen atom of the quinoline is being protonated. Additionally, the most deshielded signal upon protonation systematically corresponds to the hydrogen atom *para* to the nitrogen atom, though this only occurs with BOD 1 and BOD 2 since BOD 3 has no hydrogen atom *para* to the nitrogen atom. Interestingly, the signals close to the nitrogen atom (number 2 and 8, Fig. S1.4) start to shift upfield after the addition of 3 equivalents of acid. For BOD 2 and BOD 3, the signals of the major and minor rotamers seem to be shifted the same way upon protonation and the ratio, 1 : 2.5, remained unchanged. This indicates that protonation does not affect the equilibrium between the rotamers.

### UV-vis spectroscopy

**Fig. 5:**
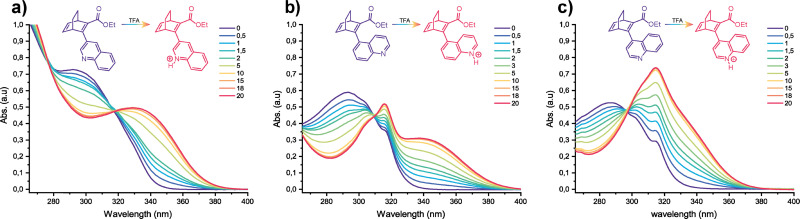
UV-vis absorption spectra recorded during the gradual addition of TFA equivalents in (a) BOD 1, (b) BOD 2 and (c) BOD 3 in acetonitrile.

**Fig. 6:**
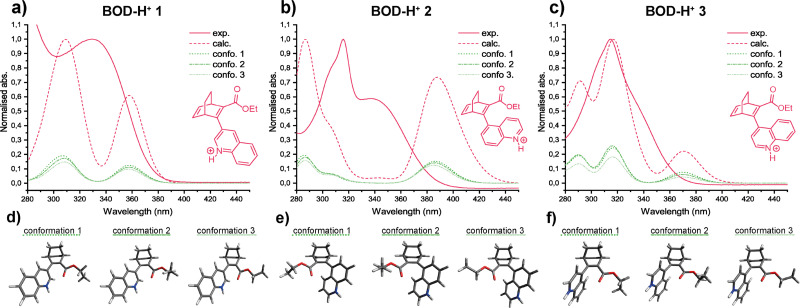
Comparison between experimental and calculated UV-vis absorption spectra in acetonitrile for (a) BOD-H^+^ 1, (b) BOD-H^+^ 2, (c) BOD-H^+^ 3 and the 3D conformations of the three most stable conformers for (d) BOD-H^+^ 1, (e) BOD-H^+^ 2, (f) BOD-H^+^ 3 (larger figures available in SI).

The UV-vis spectra of the three BODs were recorded in acetonitrile and the spectral changes were monitored upon addition of TFA (Fig. 5 and Table 4). For the three BODs, increasing acidity induces a gradual red-shift in the absorbance, reaching an equilibrium around 20 equivalents of TFA, meaning that the protonated quinoline form should have become largely prevalent after 18–20 equivalents. For BOD 1, the absorbance is simply red-shifted with the onset and the maximum shifting from 351 nm and 292 nm to 388 nm and 330 nm respectively. The protonation of BOD 2 and BOD 3 leads to more distinct spectral changes. BOD 2 does show a great redshift of the initial onset and maximum from 336 nm and 293 nm to 391 nm and 337 nm. However, it seems that the shoulder is not redshifted upon protonation but rather suffers a hyperchromic effect. BOD 3 shows a similar behaviour but while its initial onset and maximum were less red-shifted than BOD 2 (from 346 nm and 287 nm to 378 nm and approximately 335 nm, respectively), the absorbance of the shoulder increases far more than that of the shoulder of BOD 2. The reduced red-shift could be explained by the change in conjugation of the rotamer, as the quinoline unit in the minor rotamer is out of plane and therefore less conjugated with the BOD core, resulting in a smaller shift in absorbance when protonated. The experimental and calculated data were compared in the same manner as for the non-protonated state (Fig. 6). The three most stable conformers as per calculations were also assessed. Generally, the calculations show a combination of peaks that look qualitatively similar to the experimental spectra but positioned differently in the spectrum. The calculated UV-vis spectrum of BOD-H^+^ 1 is composed of two peaks which appear to be red-shifted in comparison to the experimental data, with the lowest energy band peaking at 358 nm. The calculated spectrum of BOD-H^+^ 2 is quite different from the experimental spectrum. It shows three peaks, one of which is quite red-shifted with a maximum at 387 nm and the two others being blue-shifted in comparison to the experimental spectrum. The UV-vis spectrum of BOD-H^+^ 3 has a good agreement between experiment and theory. There is a red-shifted peak with a maximum around 370 nm, another peak very close to the experimental minor rotamer at 317 nm and a final slightly blue-shifted peak. Interestingly, the calculated spectra of the lowest energy conformers of the protonated BODs are identical. This suggests that protonation would somehow narrow down the conformational freedom of the quinoline moiety making the spectra of the three most stable calculated conformations almost indistinguishable (Fig. 6d–f and Fig. S5.5–S5.7). This is against our experimental observation where shoulders corresponding to the minor rotamers are still present in the UV-vis spectra upon protonation of BOD 2 and BOD 3.

**Table 4:** Experimental and calculated absorption profiles in acetonitrile. Absorption onset (*λ*_onset_), absorption maxima (*λ*_max_) and molar extinction coefficient at the absorption maxima (*ε*_max_) for BOD-H^+^ 1, BOD-H^+^ 2 and BOD-H^+^ 3

BOD-H^+^	Experimental			Calculation
	*λ*_max_ [nm]	*ε*_max_ [M^−1^ cm^−1^]^c^	*λ*_onset_ [nm]^d^	*λ*_max_ [nm]^e^
1	330	5700	388	358
2	337	3400	391	387
—	316^a^	5770	—	—f
3	335	4600	378	370
—	315^b^	8380	—	—f

a *λ*_max_ at the shoulder in the UV-Vis spectrum of BOD 2 assigned to the minor rotamer.

b *λ*_max_ at the shoulder in the UV-Vis spectrum of BOD 3 assigned to the minor rotamer. The values are identical to the ones reported in Table 2 since the shoulder is not red-shifted by protonation.

c Calculated as *ε* = A/c considering that the *C*_BOD-H+_ = *C*_BOD_ at 20 equivalents of acid and using Abs. at *λ*_max_ since there is an overlap in absorption for the rotamer pairs.

d Calculated as log(*ε*) = 2.

e Maximum of the most red-shifted peak for each calculated spectrum.

f The identification of the maxima of the minor rotamer is not possible in the calculated spectra.

### Irradiation of BODs-H^+^

The three BODs were dissolved in acetonitrile, and 20 equivalents of acid (TFA) were added to each solution, which were then subjected to irradiation with UV light to switch from BOD-H^+^ to TCO-H^+^. Surprisingly, each BOD-H^+^ showed a different behaviour when irradiated. BOD-H^+^ 1 converts to TCO-H^+^ 1, when irradiated with a 340 nm LED, but without any major changes in the properties, the half-life of TCO-H^+^ 1 is 14.9 seconds (Fig. S4.5, *t*_1/2_ = 12.8 s for TCO 1). On the other hand, BOD-H^+^ 2 and BOD-H^+^ 3 behave differently upon UV irradiation. BOD-H^+^ 2 cannot be switched to the corresponding TCO-H^+^ 2, as no change in the UV spectrum was observed. However, we noticed that the protonated sample, BOD-H^+^ 2, is visibly fluorescent under UV light (Fig. S4.10). Fluorescence is a competitive excited-state process to photoswitching;^43^ in the case of BOD-H^+^ 2, fluorescence appears to be more efficient than photoswitching with the fluorescence quantum yield reaching 11% *versus* 3% for the photoisomerization quantum yield of neutral BOD 2. Upon UV irradiation, the absorption of BOD-H^+^ 3 slowly decreased indicating that the molecule is changing in some way. However, no thermal back conversion was observed when the irradiation was stopped. In that case, either TCO-H^+^ 3 is stuck in a “lock state” or the protonated molecule degrades upon irradiation. Further NMR and mass spectrometry studies were carried out to ascertain what is happening upon the irradiation of BOD-H^+^ 3.

BOD 3 with 20 equivalents of acid (deuterated TFA) was analysed by NMR spectroscopy in deuterated acetonitrile and irradiated with a 365 nm LED and a new spectrum was recorded every 10 minutes for 40 minutes (Fig. S2.24). During irradiation, the signals corresponding to BOD-H^+^ 3 disappear and are replaced by new signals. After 40 minutes of irradiation, the typical signals of the ethylenic hydrogen atoms of the BOD core, between 6.5 and 7 ppm, have fully disappeared. This could arise from the [2+2] cycloaddition to the TCO form, triggered by light, but it could instead arise from the degradation of BOD-H^+^ 3. The presence of weaker signals also encourages us to believe that there is more than one compound produced during the irradiation. The same experiment was carried out with 2 and 0.5 equivalents of acid for BOD-H^+^ 3 (Fig. S2.25 and S2.26) and for BOD-H^+^ 1 and BOD-H^+^ 2 with 20 equivalents (Fig. S2.22 and S2.23).

For BOD-H^+^ 1 and BOD-H^+^ 2, no change was observed after 20 minutes of irradiation as expected since the former has a photoisomer with a very short half-life and the latter does not switch because of competitive fluorescence. However, after addition of a base (20 equivalents of triethylamine, TEA), it seems that both molecules return to the non-protonated state proving that the acid–base equilibrium between BOD and BOD-H^+^ exists.

**Fig. 7:**
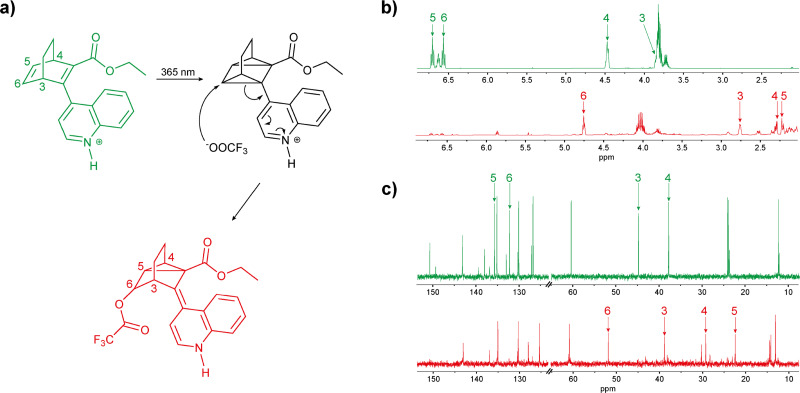
(a) Proposed mechanism for the degradation of BOD-H^+^ 3 upon irradiation in the UV in acetonitrile in the presence of TFA and comparison between the NMR spectra of BOD-H^+^ 3 before (green) and after (red) irradiation in (b) ^1^H and (c) ^13^C.

For BOD-H^+^ 3, marginal differences are observed between 2 and 20 equivalents when the sample is irradiated. However, the sample with 0.5 equivalents of acid did not fully convert and no changes were observed after 20 minutes of irradiation. This indicates that there are two conditions for the sample to degrade: being irradiated in the UV as well as being fully protonated. To prove that the phenomenon observed is irreversible, the samples with 2 and 20 equivalents after irradiation were heated at 40 °C for three days (Fig. S2.27). After three days of heating, no changes were observed in the NMR spectra revealing the irreversibility of the conversion. In a separate experiment, a sample of BOD-H^+^ 3 with 20 equivalents of acid was irradiated then 20 equivalents of triethylamine were added to see if the addition of base would return BOD 3 (Fig. S2.28). However, the new signals that appeared throughout the irradiation were simply shifted, and it seems that no BOD 3 was recovered. This is another proof that BOD-H^+^ 3 most probably degrades in some way during the irradiation. To elucidate the possible degradation of BOD-H^+^ 3, NMR and MS analyses were performed. In LC-MS, additional signals are detected upon irradiation at *m/z* 420 with exact mass corresponding to [BOD 3 + CF_3_COOH + H]^+^ (Fig. S3.1 and Table S3-1). Their abundance also changed according to the number of equivalents of acid in solution, which matches what was previously observed during the irradiation of BOD-H^+^ 3 with different equivalents of acid in ^1^H-NMR. Also, ^19^F-NMR was used to compare the spectra of BOD-H^+^ 3 before irradiation and after irradiation (Fig. S2.29). Surprisingly, new signals appear in the ^19^F-NMR spectrum, meaning that new compounds containing fluorine atoms are being generated. These observations in LC-MS and ^19^F-NMR would suggest that the trifluoroacetate reacts with the protonated photoisomer since this degradation is solely observed when enough equivalents of acid are present and upon irradiation with UV light. With this in hand, a mechanism of such a reaction is proposed and shown in Fig. 7.

It is believed that this mechanism is favoured by the possibility of neutralising the charge on the quinoline while also releasing some strain by breaking one of the three-membered rings in the TCO. The former explanation also demonstrates why this degradation is observed only during the irradiation of BOD-H^+^ 3; the position of the nitrogen allows the charge to be neutralised through conjugation which would not be possible in BOD-H^+^ 1 and BOD-H^+^ 2. The proposed structure is also consistent with the NMR and MS analyses (for NMR Fig. S2.30–S2.37, for MS Fig. S3.2 and S3.3). Fig. 7b and c shows the changes in the NMR spectra of protons and carbons 3, 4, 5 and 6 before and after the degradation, where the signals corresponding to nuclei 5 and 6 are clearly shifting from the ethylenic region to the alkane one. Also, it is known that there are at least two major degradation products since there are 2 major signals in both ^19^F-NMR and LC-MS. However, the characterisation of the second product is more difficult because the signals are subtle in NMR.

## Conclusion

We successfully synthesised three different quinoline-substituted BOD isomers through a Diels–Alder reaction and a Suzuki coupling. Two of these isomers, BOD 2 and BOD 3, were isolated as a pair of conformational rotamers. We studied this rotamerism behaviour extensively experimentally with NMR and UV-vis analyses as well as computationally. The substituents allowed all the BODs to have their absorption red-shifted by approximately 120 nm when compared with the calculated spectrum of unsubstituted BOD.^44^ The three isomers were successfully photoswitched to the corresponding TCOs upon irradiation at 308 nm with little to no degradation observed within several cycles. However, the spectral changes during the switching are negligible due to the low quantum yields of photoconversion for each BOD as well as the quick back conversion from TCO to the BOD form. The thermal half-lives of the TCOs were found to be modified by the substitution position of the quinoline, increasing from seconds to minutes from BOD 1 (12.8 s) to BOD 3 (144.6 s) and BOD 2 (382.8 s), attributed to increased steric crowding in BOD 2 and BOD 3. We studied the impact of protonation of the quinoline moiety which proved to change the photophysical properties of the BODs. The absorptions of all the BODs were red-shifted by ≈ 40 nm with the addition of acid. Unexpectedly, we could not trigger the photoswitching for any protonated BODs and each of them exhibited a different behaviour upon irradiation with UV light. BOD-H^+^ 1 could be converted to the corresponding TCO-H^+^ 1 but without a notable change in the half-life. BOD-H^+^ 2 did not photoswitch but was found to be fluorescent, a behaviour that competes with photoisomerization. It emits light around 500 nm with a fluorescence quantum yield reaching 11%. Finally, we proved with several experiments in NMR and MS that BOD-H^+^ 3 is degrading upon irradiation through an addition mechanism involving the trifluoroacetate from the TFA and the TCO-H^+^. We would like to note that while the BOD/TCO system needs improvements in storage time and quantum yield, this study has shown that using substituents with a protonation site can lead to critical changes in the photophysical properties of a photoswitch as well as completely modifying the behaviour towards irradiation with the sole position of the nitrogen atom in our case. We envision that the experimental and computational results presented here can help to further understand the properties of future heterocycle-substituted photoswitches.

## Author contributions

P. A. G. main author and responsible for writing the original draft, performed the synthesis and characterisation of the molecules by NMR spectroscopy, UV-vis spectroscopy, cyclisation study, kinetic study of the back-conversion and quantum yields measurements. J. L. E. second author carried out all computational works and helped with the experiments involving the in-house automated set-up. R. J. S. assisted with the experiments involving the protonation of the BODs through addition of acid as well as the writing of the draft. P. F. helped with the identification of the degradation product from the irradiation of BOD-H^+^ 3 and with the NMR spectra analysis. T. R. performed all the mass spectrometry analyses. P. G. and K. M. P. corresponding authors supervised the project and are responsible for funding acquisition.

## Conflicts of interest

There are no conflicts of interest.

## Supplementary Material

CP-028-D6CP00936K-s001

## Data Availability

The data supporting this article has been included as part of the supplementary information (SI). Supplementary information is available. See DOI: https://doi.org/10.1039/d6cp00936k.
