# Supplementary material for: Photoswitching behaviour of quinoline-substituted bicyclooctadienes and the role of protonation
Source: Phys Chem Chem Phys. 2026 Apr 14;28(17):10581–90. doi: 10.1039/d6cp00936k (PMC13077278; doi:10.1039/d6cp00936k)
Supplement: CP-028-D6CP00936K-s001 [file CP-028-D6CP00936K-s001.pdf]

Supplementary Information (SI) for Physical Chemistry Chemical Physics.
This journal is © the Owner Societies 2026

Photoswitching behaviour of quinoline-substituted bicyclooctadienes and the role of protonation

Supporting information

Table of Contents

Supporting information 1

1. Molecules and synthesis 2
2. NMR analysis 4
3. Mass spectrometry 25
4. UV-vis spectroscopy 27

Molar extinction coefficient 27

Kinetics of the back conversion 28

Cyclability study 30

Quantum yields measurements 31

Photoswitching 32

5. Theoretical methods 32

1. Molecules and synthesis

- <u>ethyl-3-bromopropiolate</u>

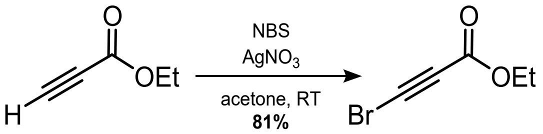

Figure S1.1: Synthesis of ethyl-3-bromopropiolate

In a degassed round bottomed flask containing the solvent (acetone, 15mL), ethyl propiolate (20 mmol, 1 equiv.), NBS (22 mmol, 1.1 equiv.) and AgNO3 (2 mmol, 0.1 equiv.) are added. The mixture is stirred at room temperature for 3 hours covered from sunlight with aluminium foil.

After 3 hours, the mixture is filtered through a silica pad then extracted with DCM and water, the organic layers are then gathered. Rotatory evaporation of the solvent afforded the desired product with a yield of 81%.

^{1}H NMR (400 MHz, Chloroform-*d*) δ 4.25 (q, *J* = 7.1 Hz, 2H), 1.32 (t, *J* = 7.1 Hz, 3H).

- <u>ethyl-3-bromobicyclo[2.2.2]octa-2,5-diene-2-carboxylate</u>

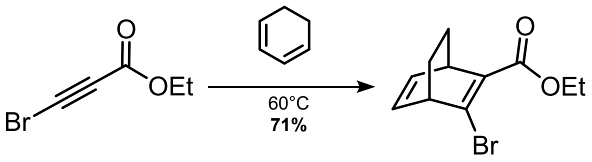

Figure S1.2: Synthesis of ethyl-3-bromobicyclo[2.2.2]octa-2,5-diene-2-carboxylate

Ethyl-3-bromopropiolate (16 mmol, 1 equiv.) and 1,3-cyclohexadiene (19 mmol, 1.2 equiv.) are mixed into a sealed microwave flask then heated at 60°C while stirring for 4 days.

Purification of the product was carried out via flash chromatography (EtOAc in petroleum spirit) and the product was isolated with a yield of 71%.

^{1}H NMR (400 MHz, Chloroform-*d*) δ 6.37 (ddd, *J* = 7.4, 6.0, 1.5 Hz, 1H, ***H***RC=CRH), 6.31 (ddd, *J* = 7.4, 5.9, 1.9 Hz, 1H, HRC=CR***H***), 4.33 (m, 1H, ***H***R$_3$C), 4.24 (qq, *J* = 7.2, 3.7 Hz, 2H, COOC***H$_2$***CH$_3$), 3.91 (m, 1H, ***H***R$_3$C), 1.65 – 1.57 (m, 1H, R***H$_2$***C-CH$_2$R), 1.52 – 1.44 (m, 1H, R***H$_2$***C-CH$_2$R), 1.42 – 1.31 (m, 5H, RH$_2$C-C***H$_2$***R and COOCH$_2$C***H$_3$***).

- <u>General procedure for the Suzuki coupling using quinoline boronic acid</u>

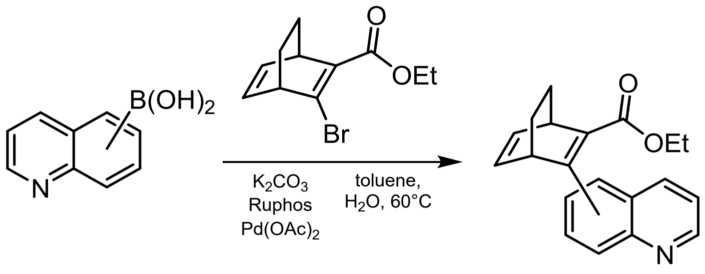

Figure S1.3: Suzuki coupling using quinoline boronic acid

In a degassed flask, ethyl-3-bromobicyclo[2.2.2]octa-2,5-diene-2-carboxylate (1 equiv.) was added along with dry toluene (~25mL) and deionised water (~6mL) under nitrogen flow. Then potassium carbonate (4.6 equiv.), Pd(OAc)$_2$ (0.05 equiv.), Ruphos (0.1 equiv.) and the crude from the borylation are added to the flask. The biphasic mixture is then stirred at 60°C for 2 days under nitrogen atmosphere.

After cooling at room temperature, the mixture is filtered through a silica plug and quenched with 20 mL of a saturated NH_4Cl solution as well as 20 mL of deiosined water. The mixture is then extracted with toluene (3 x 20 mL) and the organic layers are gathered and dried over $MgSO_4$ then filtered and rotatory evaporation is used to remove the solvent.

The product is then purified via flash chromatography (EtOAc in petroleum spirit). With this protocol the synthesis of **BOD 1**, **BOD 2** and **BOD 3** were successful with yields of 29 %, 26 % and 24 % respectively.

<u>Analysis of **BOD 1**:</u>

^{1}H NMR (400 MHz, Chloroform-d) δ 8.73 (d, J = 2.2 Hz, 1H, Ar-***H***), 8.10 (d, J = 8.5 Hz, 1H, Ar-***H***), 8.01 (d, J = 1.4 Hz, 1H, Ar-***H***), 7.81 (dd, J = 8.2, 1.4 Hz, 1H, Ar-***H***), 7.71 (ddd, J = 8.4, 6.9, 1.5 Hz, 1H, Ar-***H***), 7.55 (ddd, J = 8.1, 6.9, 1.2 Hz, 1H, Ar-***H***), 6.55 (ddd, J = 7.5, 6.1, 1.6 Hz, 1H, ***H***RC=CRH), 6.47 (ddd, J = 7.4, 5.9, 1.6 Hz, 1H, HRC=CR***H***), 4.43 – 4.36 (m, 1H, ***H***R$_3$C), 4.03 (qd, J = 7.1, 1.7 Hz, 2H, COOC***H$_2$***CH$_3$), 3.98 – 3.93 (m, 1H, ***H***R$_3$C), 1.69 – 1.59 (m, 2H, R***H$_2$***C-CH$_2$R), 1.55 – 1.45 (m, 2H, RH$_2$C-C***H$_2$***R), 0.97 (t, J = 7.1 Hz, 3H, COOCH$_2$C***H$_3$***).

^{13}C NMR (101 MHz, $CDCl_3$) δ 165.41, 152.45, 149.31, 145.64, 135.45, 134.98, 132.98, 132.75, 130.15, 128.21, 127.93, 127.52, 127.31, 60.52, 46.05, 38.80, 24.84, 24.65, 13.90.

HRMS: C20H20NO2$^+$, *m/z* 306,1495 measured (0,3 ppm), *m/z* 306,1494 calculated

<u>Analysis of **BOD 2**, rotamer signal are specified by ***H***^{r}**:**</u>

^{1}H NMR (400 MHz, Chloroform-*d*) δ 8.90 (dt, *J* = 4.6, 2.3 Hz, 1.4H, Ar-***H*** and Ar-***H***^{r}), 8.08 (dd, *J* = 8.6, 3.9 Hz, 1.4H, Ar-***H*** and Ar-***H***^{r}), 7.91 (d, *J* = 8.4 Hz, 0.4H, Ar-***H***^{r}), 7.87 (dd, *J* = 8.4, 1.6 Hz, 1H, Ar-***H***), 7.70 (m, 1.4H, Ar-***H*** and Ar-***H***^{r}), 7.38 – 7.29 (m, 1.8H, Ar-***H*** and 2 Ar-***H***^{r}), 7.23 (dd, *J* = 7.0, 1.2 Hz, 1H, Ar-***H***), 6.63 (ddd, *J* = 7.6, 6.2, 1.5 Hz, 1H, ***H***RC=CRH), 6.60 – 6.53 (m, 0.8H, ***H***^{r}RC=CR***H***^{r}), 6.50 (ddd, *J* = 7.4, 5.8, 1.6 Hz, 1H, HRC=CR***H***), 4.49 – 4.40 (m, 1.4H, ***H***R$_3$C and ***H***^{r}R$_3$C), 3.86 – 3.68 (m, 3.6H, ***H***R$_3$C, ***H***^{r}R$_3$C and COOC***H$_2$***CH$_3$), 3.65 (q, *J* = 7.1 Hz, 0.8H, COOC***H$_2$***^{r}CH$_3$), 1.81 – 1.47 (m, 6.2H, R***H$_2$***C-C***H$_2$***R and R***H$_2$***^{r}C-C***H$_2$***^{r}R), 0.59 (t, *J* = 7.1 Hz, 3H, COOCH$_2$C***H$_3$***), 0.39 (t, *J* = 7.1 Hz, 1.2H, COOCH$_2$C***H$_3$***^{r}).

^{13}C NMR (101 MHz, $CDCl_3$) δ 165.25, 154.86, 153.04, 149.28, 147.07, 139.53, 139.45, 136.39, 136.02, 135.67, 135.63, 135.38, 134.37, 133.23, 132.62, 129.44, 129.36, 127.90, 127.70, 126.59, 126.21, 124.69, 124.12, 121.03, 120.82, 59.94, 46.76, 46.26, 38.29, 38.21, 25.14, 24.90, 24.73, 24.38, 13.36, 13.08.

HRMS: C20H20NO2$^+$, *m/z* 306,1498 measured (1,3 ppm), *m/z* 306,1494 calculated

<u>Analysis of **BOD 3**, rotamer signal are specified by ***H***^{r}:</u>

^{1}H NMR (400 MHz, Chloroform-*d*) δ 8.89 (d, *J* = 4.5 Hz, 0.4H, Ar-***H***^{r}), 8.86 (d, *J* = 4.5 Hz, 1H, Ar-***H***), 8.20 (d, *J* = 8.5 Hz, 1.4H, Ar-***H*** and Ar-***H***^{r}), 7.73 (ddd, *J* = 8.4, 5.5, 2.8 Hz, 1.4H, Ar-***H*** and Ar-***H***^{r}), 7.58 (d, *J* = 8.3 Hz, 0.4H, Ar-***H***^{r}), 7.54 – 7.46 (m, 2.4H, 2 Ar-***H*** and Ar-***H***^{r}), 7.16 (d, *J* = 4.5 Hz, 0.4H, Ar-***H***^{r}), 7.12 (d, *J* = 4.5 Hz, 1H, Ar-***H***), 6.65 (ddd, *J* = 7.5, 6.2, 1.5 Hz, 1H, ***H***RC=CRH), 6.62 – 6.54 (m, 0.8H, ***H***^{r}RC=CR***H***^{r}), 6.51 (ddd, *J* = 7.3, 5.9, 1.5 Hz, 1H, HRC=CR***H***), 4.51 – 4.42 (m, 1.4H, ***H***R$_3$C and ***H***^{r}R$_3$C), 3.88 – 3.58 (m, 4.6H, ***H***R$_3$C, ***H***^{r}R$_3$C, COOC***H$_2$***CH$_3$ and COOC***H$_2$***^{r}CH$_3$), 1.83 – 1.48 (m, 6.4H, R***H$_2$***C-C***H$_2$***R and R***H$_2$***^{r}C-C***H$_2$***^{r}R), 0.60 (t, *J* = 7.1 Hz, 3H, COOCH$_2$C***H$_3$***), 0.39 (t, *J* = 7.1 Hz, 1.2H, COOCH$_2$C***H$_3$***^{r}).

^{13}C NMR (101 MHz, $CDCl_3$) δ 164.85, 164.73, 153.43, 151.54, 148.68, 137.18, 135.95, 135.90, 135.83, 133.15, 132.56, 130.02, 129.84, 128.82, 127.03, 126.93, 126.50, 126.08, 125.81, 125.04, 118.44, 117.94, 60.23, 45.90, 45.54, 38.10, 38.05, 25.01, 24.90, 24.66, 24.58, 13.26, 12.94.

HRMS: C20H20NO2$^+$, *m/z* 306,1495measured (0,3 ppm), *m/z* 306,1494 calculated

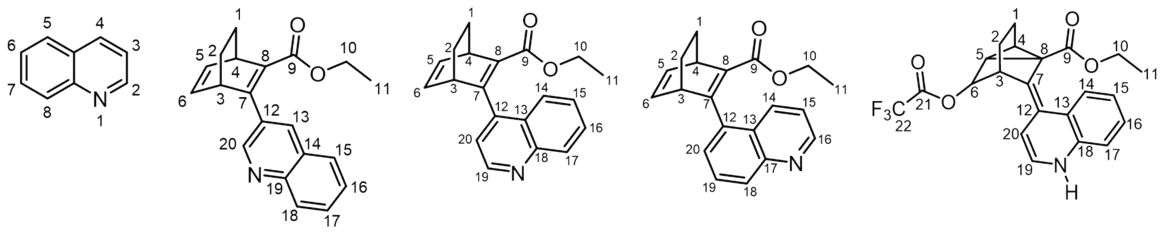

Figure S1.4: atom numbering in quinoline, the three BODs and the potential degradation product

2. NMR analysis

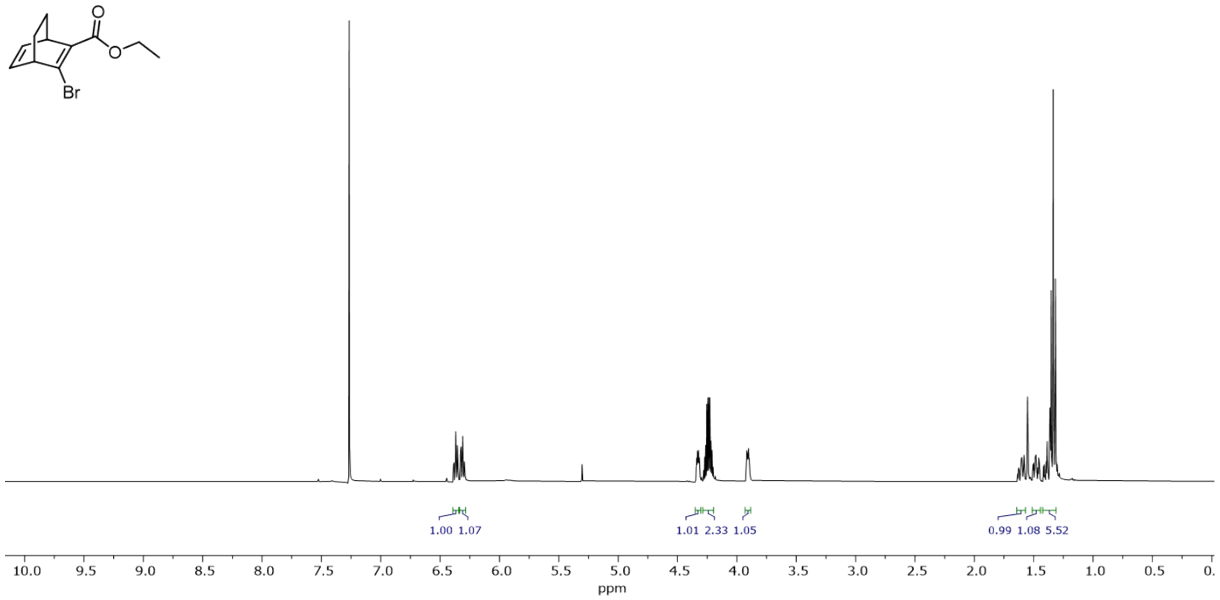

Figure S2.1: ^{1}H-NMR of ethyl-3-bromobicyclo[2.2.2]octa-2,5-diene-2-carboxylate in $CDCl_3$

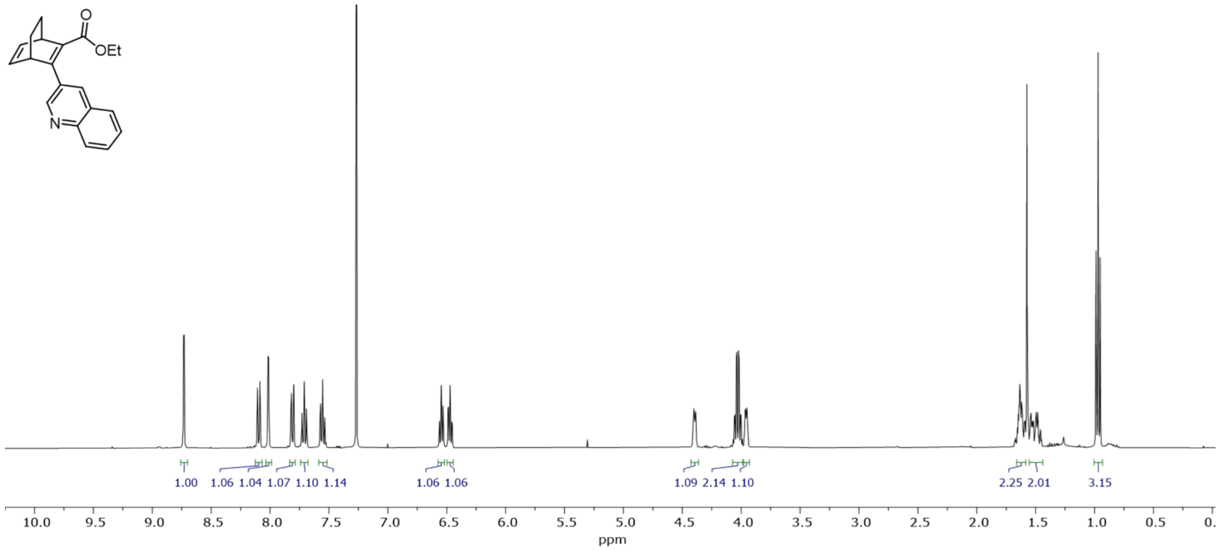

Figure S2.2: ^{1}H-NMR of BOD 1 in $CDCl_3$

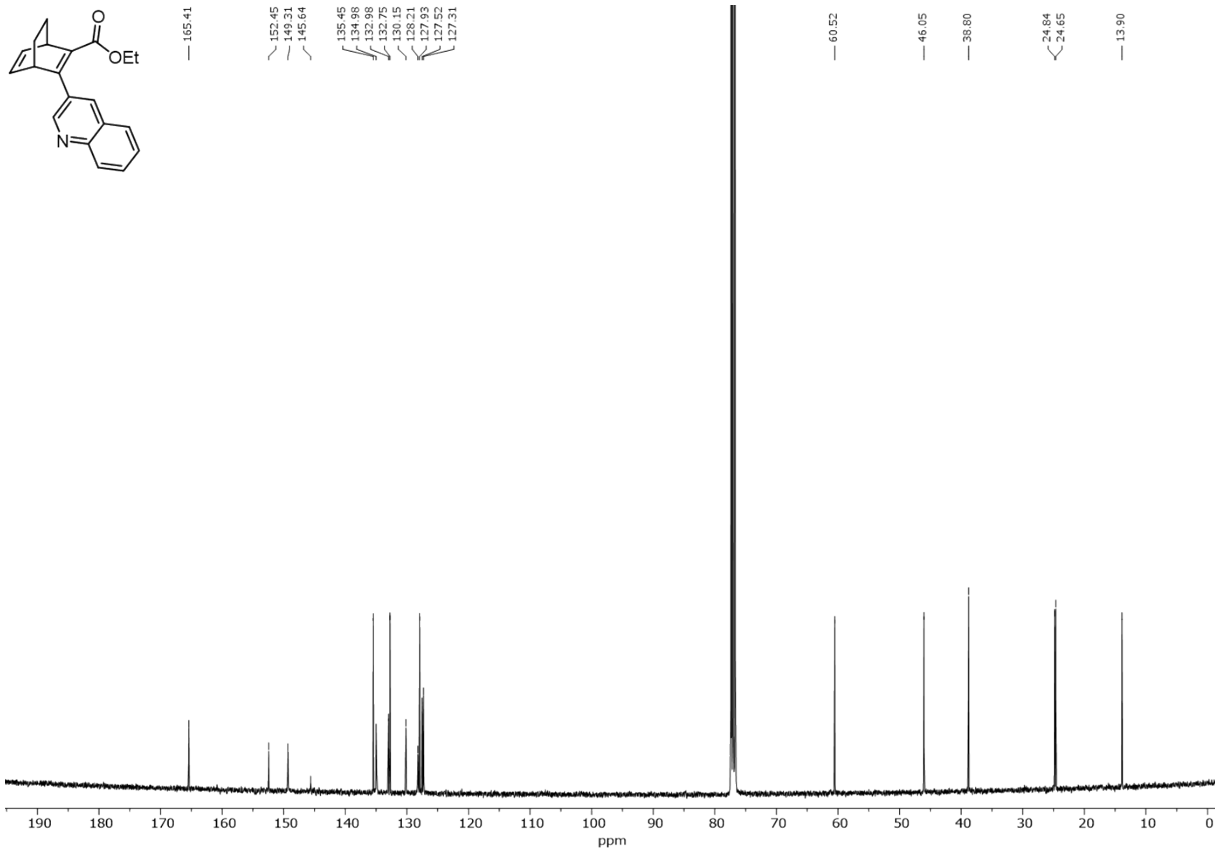

Figure S2.3: ^{13}C-NMR of BOD 1 in $CDCl_3$

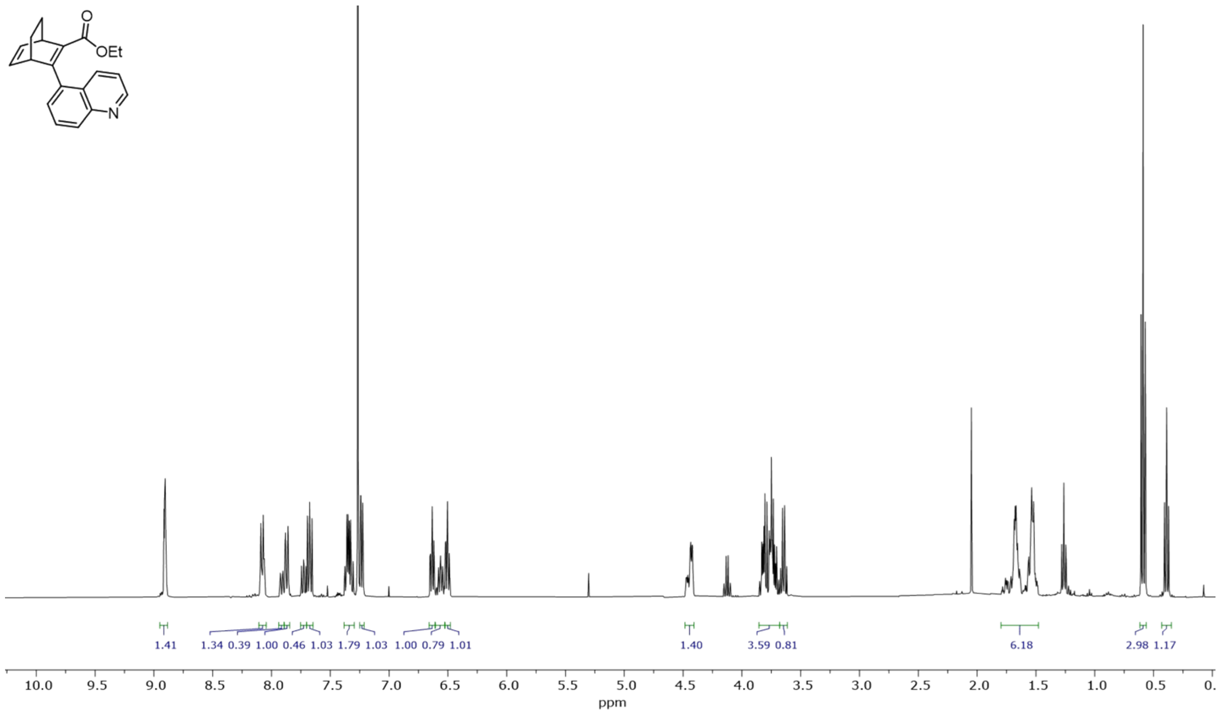

Figure S2.4: ^{1}H-NMR of BOD 2 in $CDCl_3$

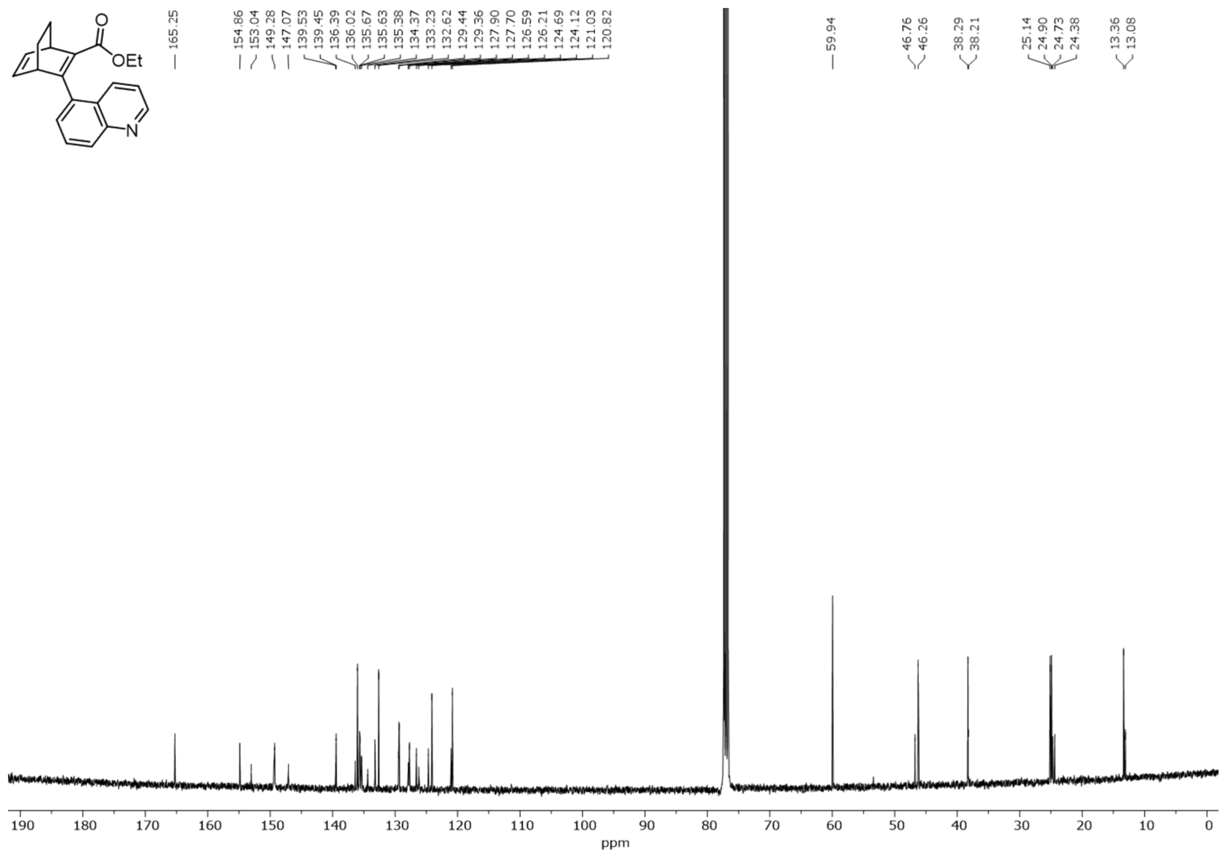

Figure S2.5: ^{13}C-NMR of BOD 2 in $CDCl_3$

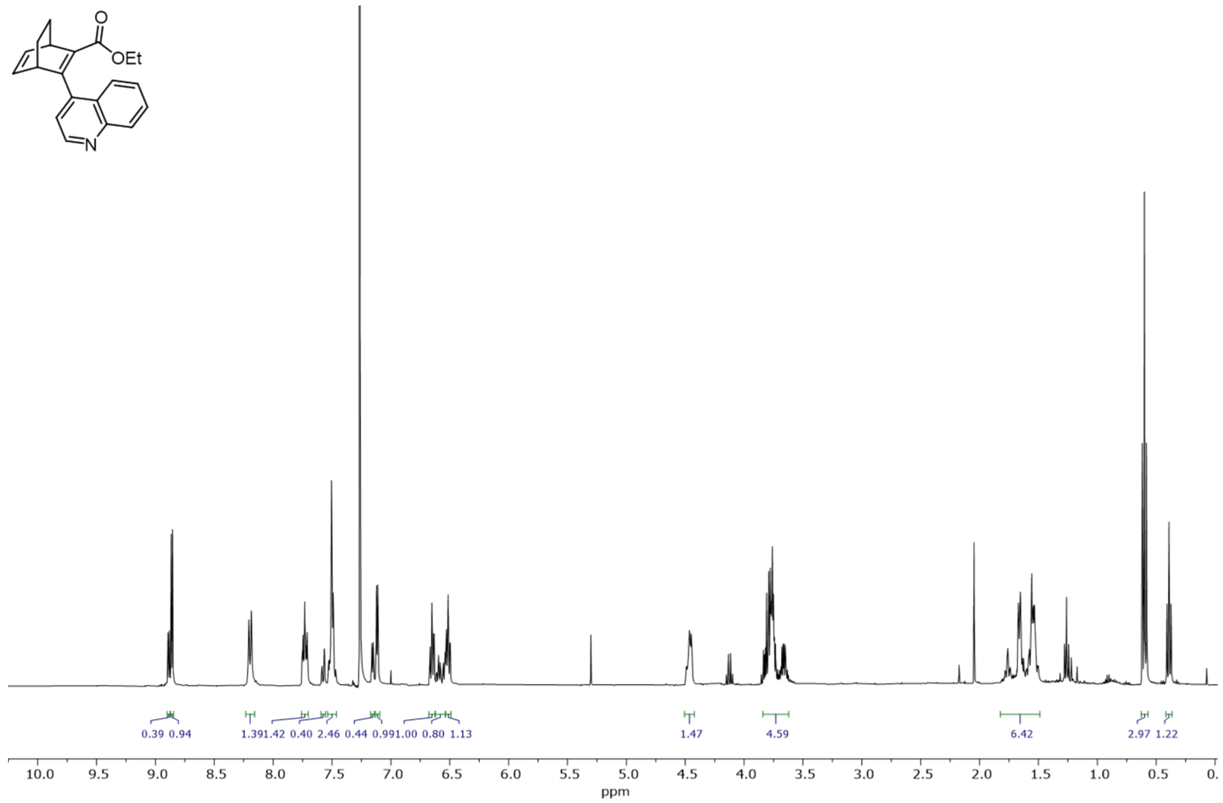

Figure S2.6: ^{1}H-NMR of BOD 3 in $CDCl_3$

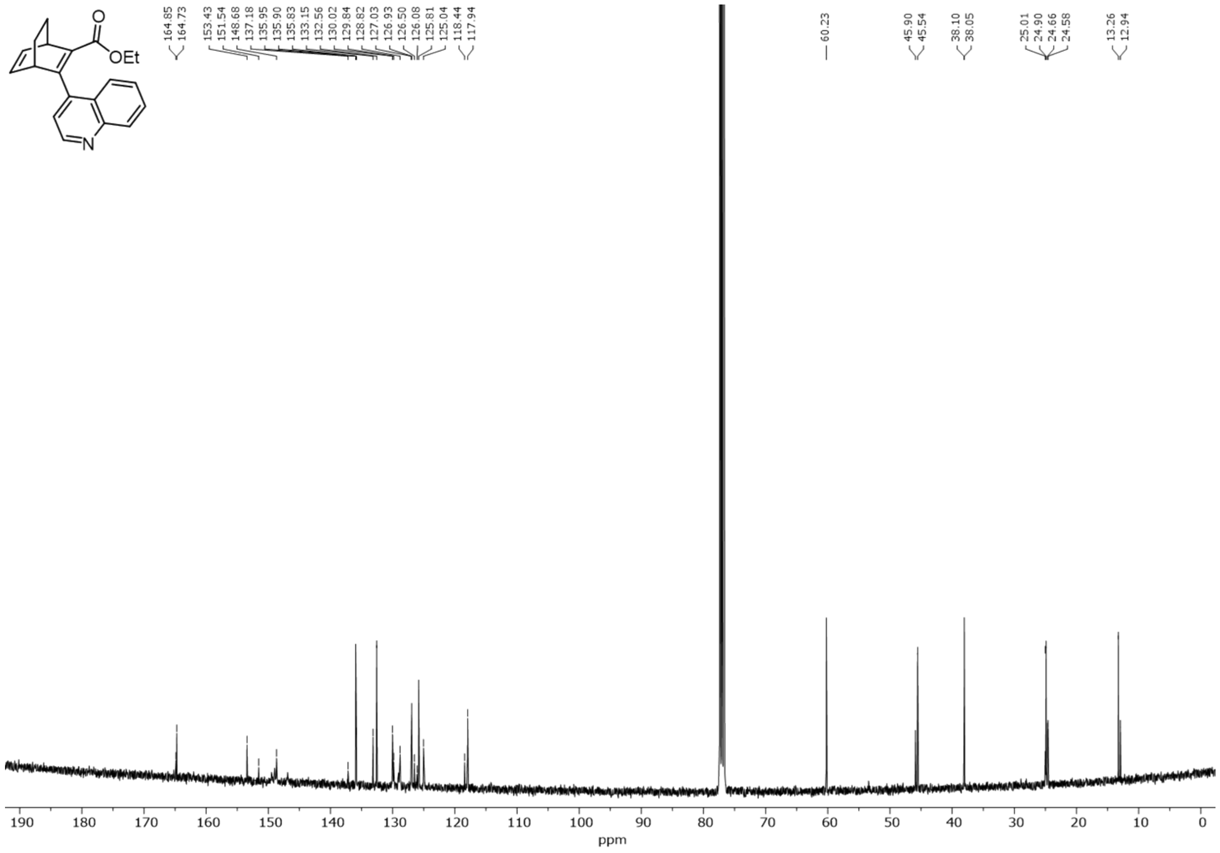

Figure S2.7: ^{13}C-NMR of BOD 3 in $CDCl_3$

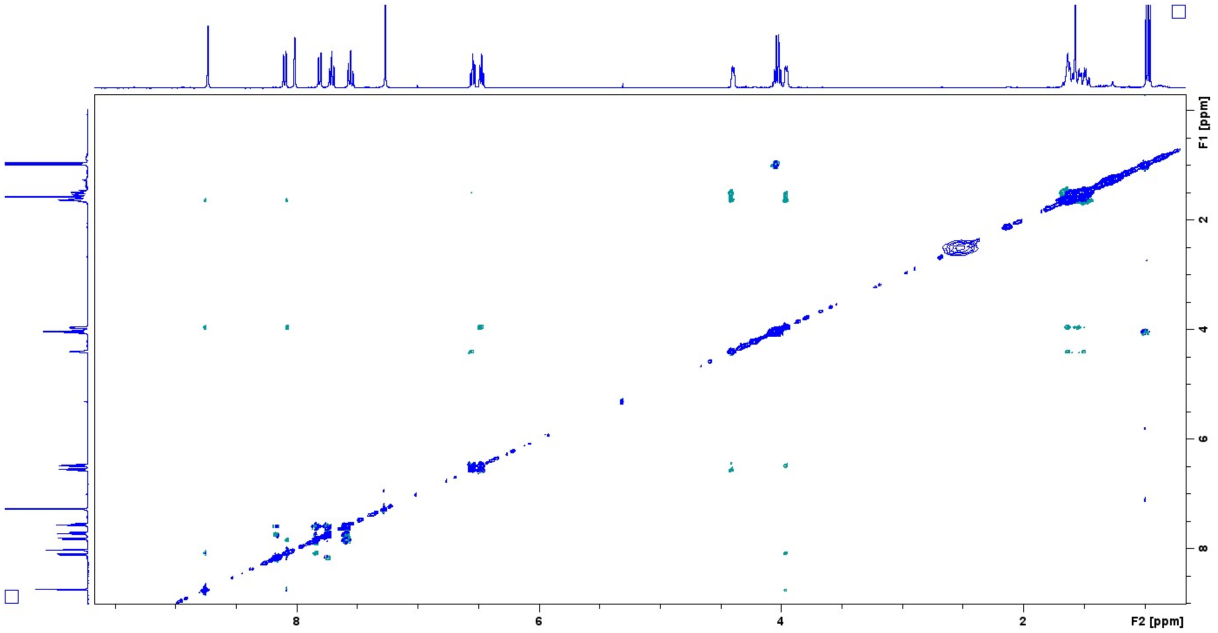

Figure S2.8 : NOESY NMR of BOD 1 in $CDCl_3$

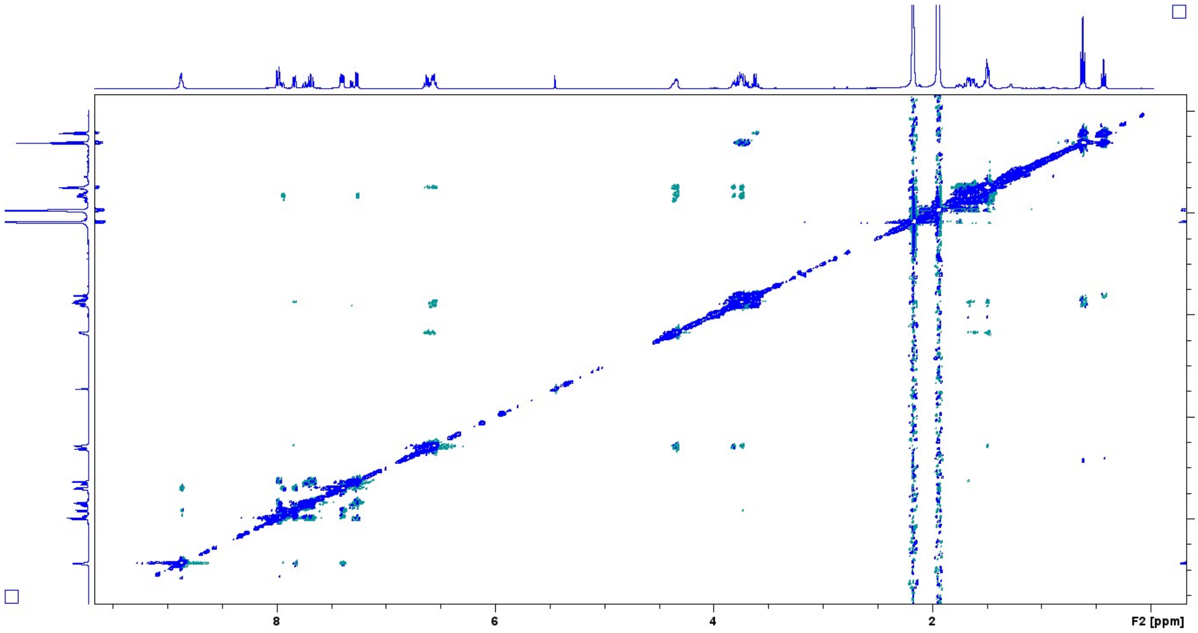

Figure S2.9: NOESY NMR of BOD 2 in CD_3CN

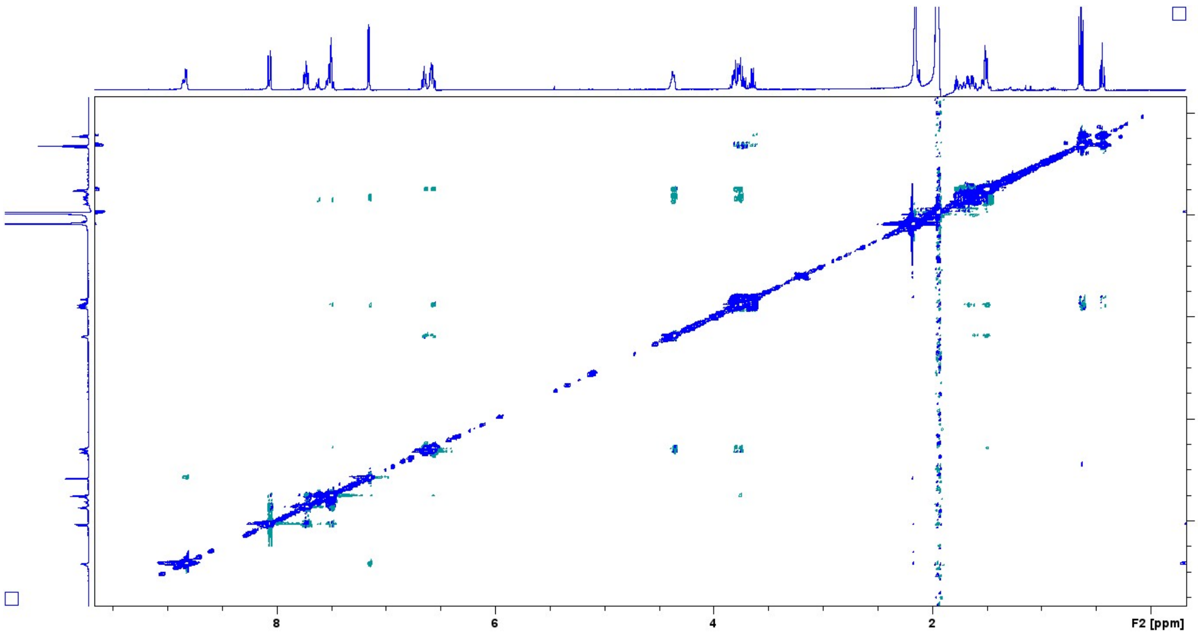

Figure S2.10: NOESY NMR of BOD 3 in CD_3CN

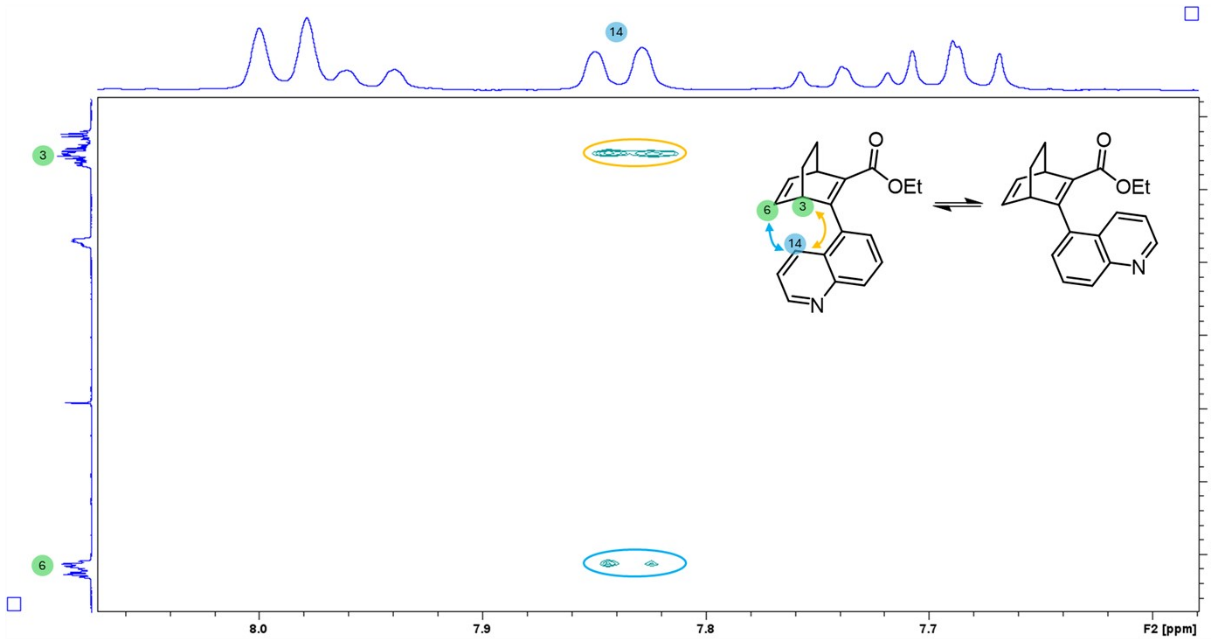

Figure S2.11: NOE signals between H-14 and H-6 and H-3 in the NOESY NMR of BOD 2

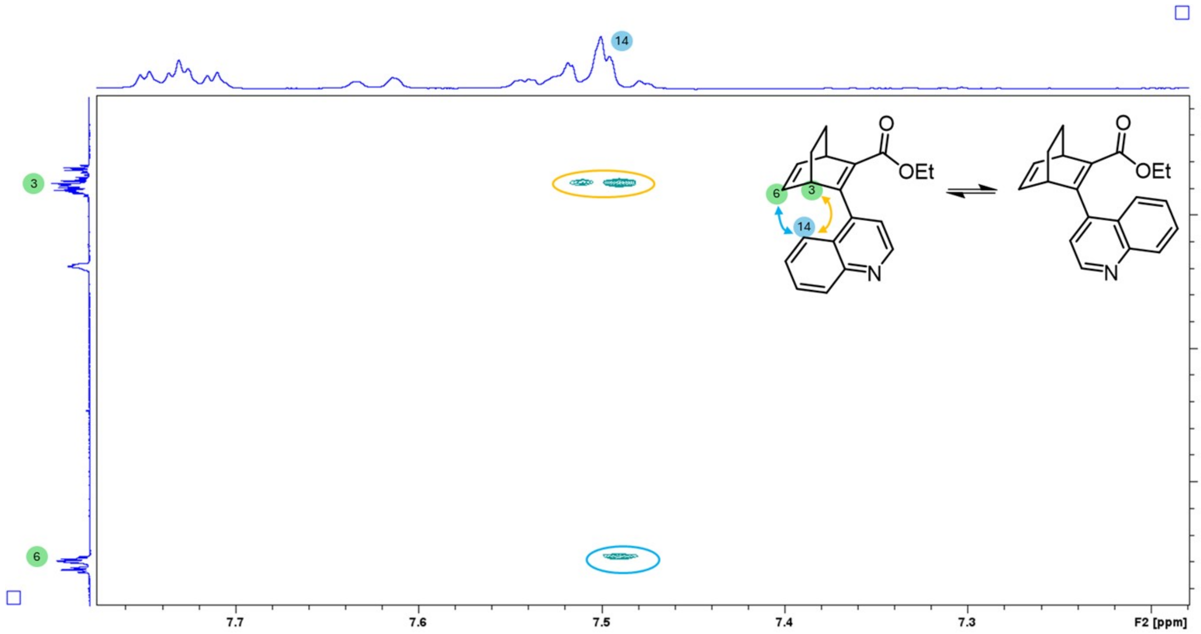

Figure S2.12: NOE signals between H-14 and H-6 and H-3 in the NOESY NMR of BOD 3

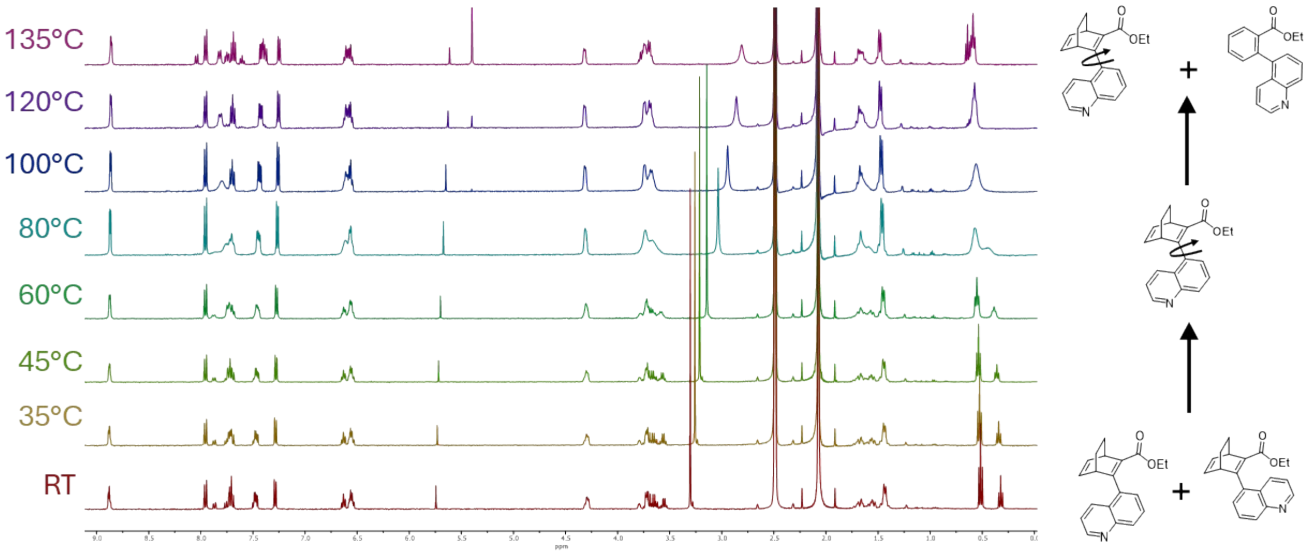

Figure S2.13: Varying temperature NMR (Heating) of BOD 2 in DMSO-d6

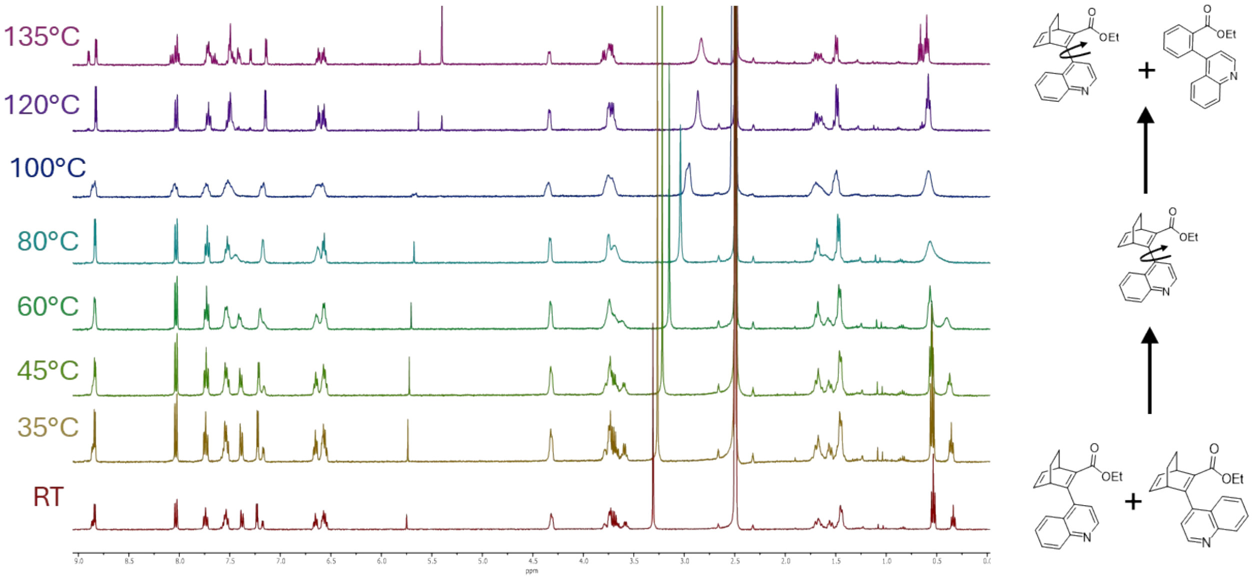

Figure S2.14: Varying temperature NMR (Heating) of BOD 3 in DMSO-d6

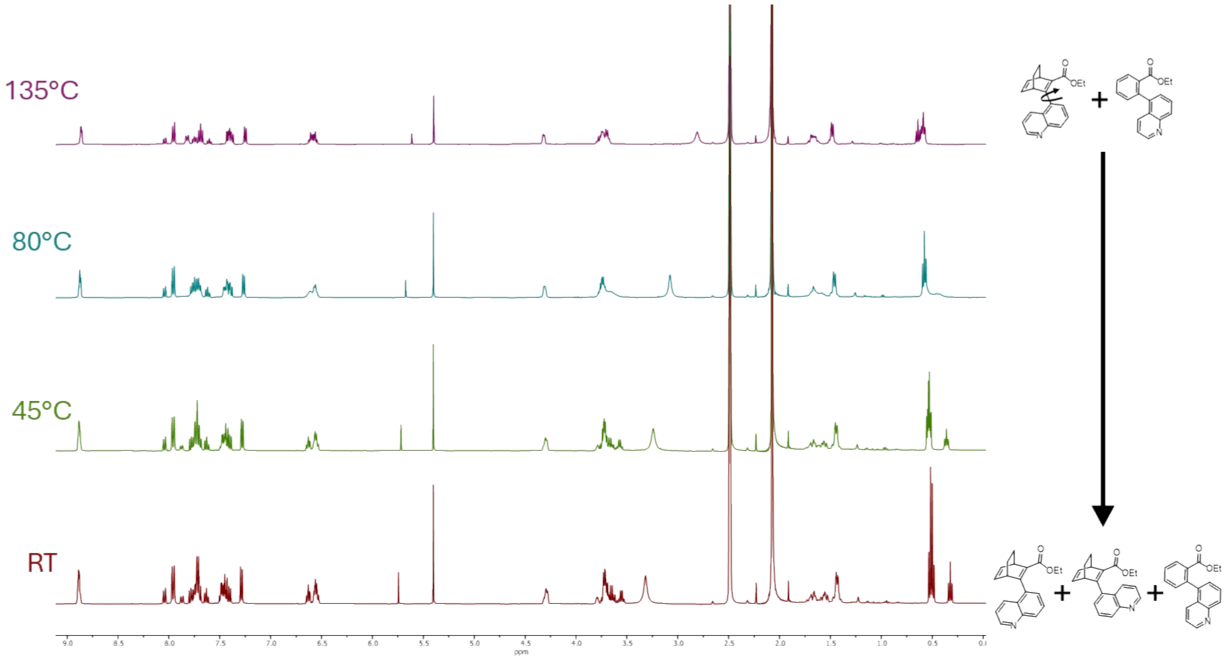

Figure S2.15: Varying temperature NMR (Cooling) of BOD 2 in DMSO-d6

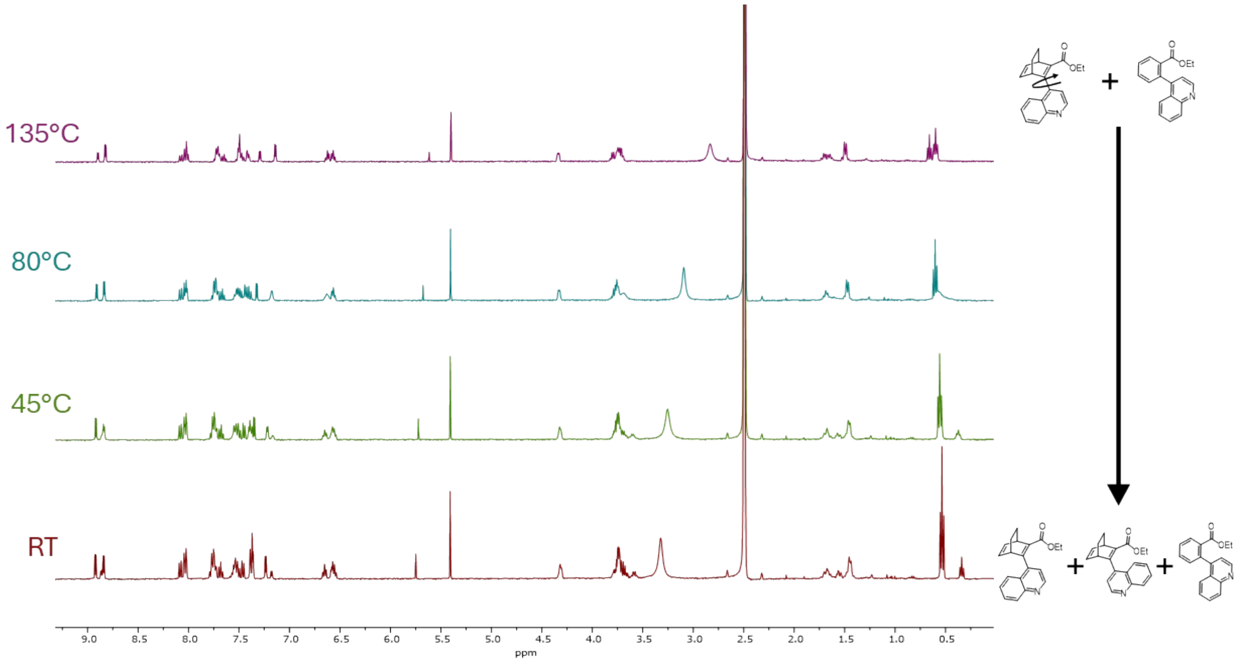

Figure S2.16: Varying temperature NMR (Cooling) of BOD 3 in DMSO-d6

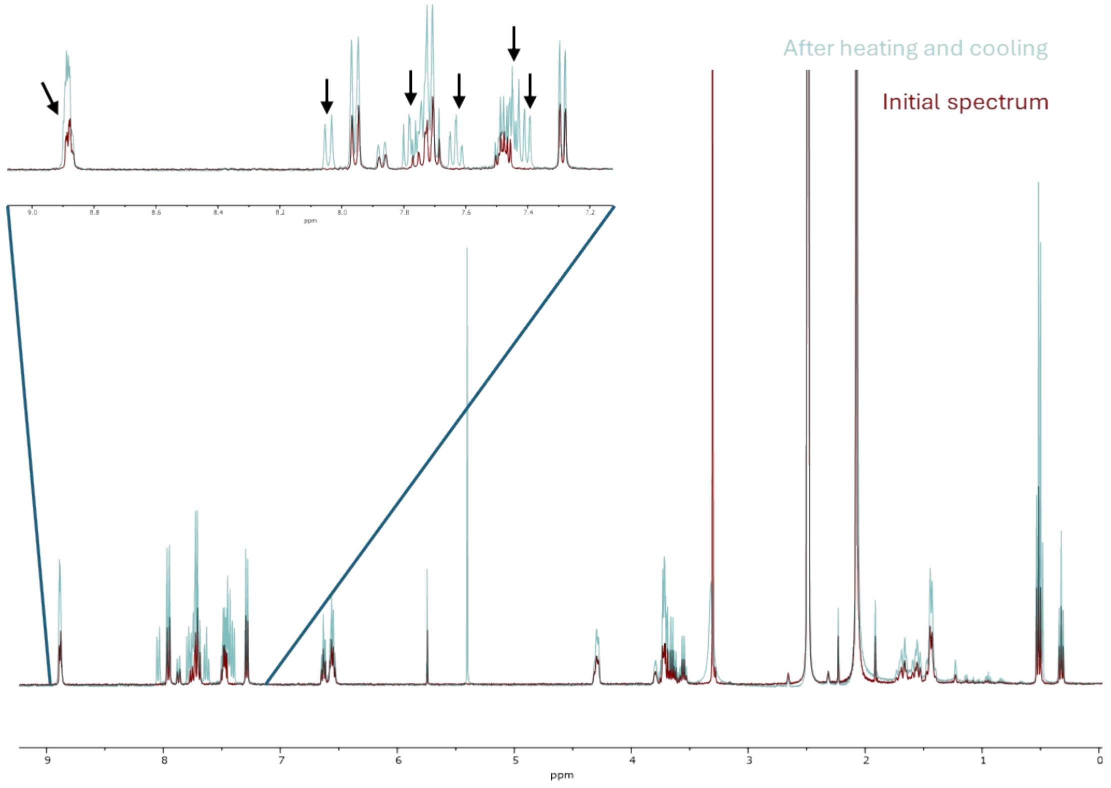

Figure S2.17: Appearance of new signals corresponding to the degradation product after heating and cooling BOD 2

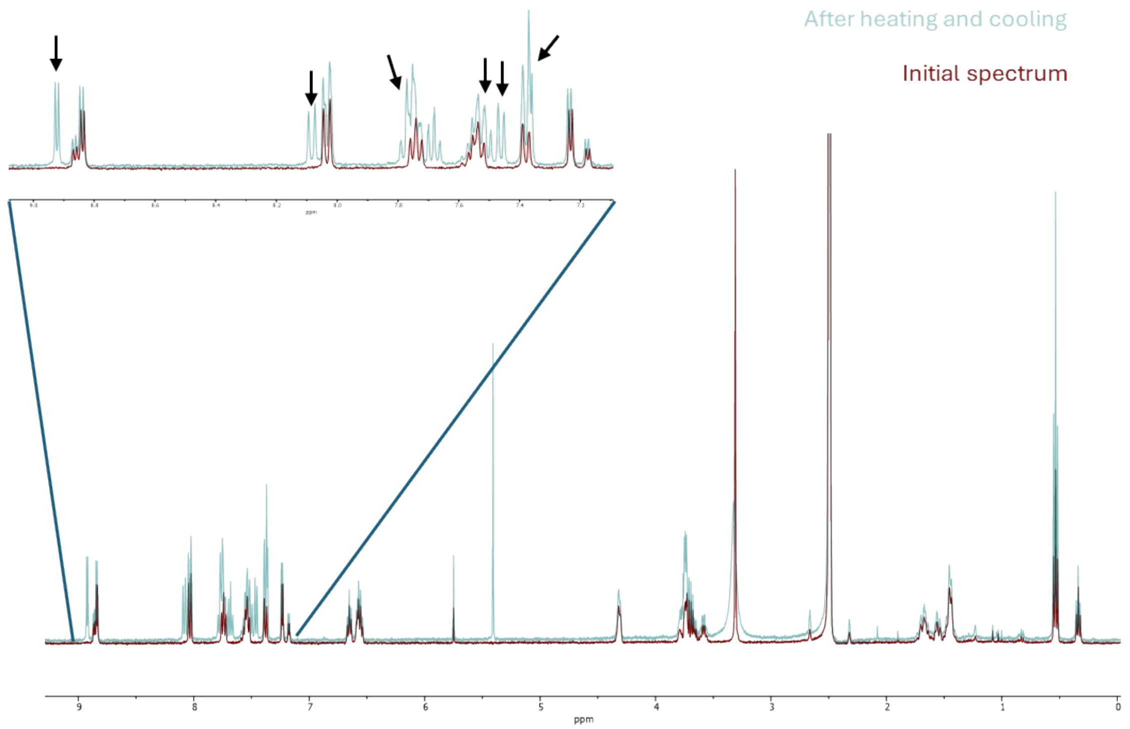

Figure S2.18: Appearance of new signals corresponding to the degradation product after heating and cooling BOD 3

$$k_{exch} = \frac{\pi(\upsilon_1 - \upsilon_2)}{\sqrt{2}}$$

$$\Delta G^{rota} = -RT_c \ln\left(\frac{k_{exch}h}{k_B T_c}\right)$$

BOD 2

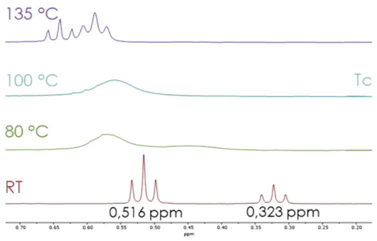

$$k_{exch} = \frac{\pi(0{,}516\ ppm - 0{,}323\ ppm) * 400\ Hz/ppm}{\sqrt{2}}$$

$$= 171{,}384\ Hz$$

$$T_c = 100\ °C = 373{,}15\ K$$

$$\Delta G_2^{rota} = 76{,}13\ kJ/mol$$

BOD 3

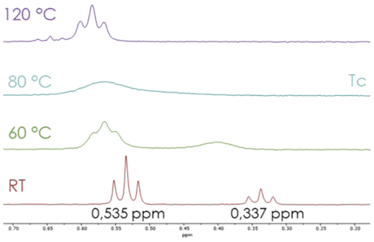

$$k_{exch} = \frac{\pi(0{,}535\ ppm - 0{,}337\ ppm) * 400\ Hz/ppm}{\sqrt{2}}$$

$$= 175{,}824\ Hz$$

$$T_c = 80\ °C = 353{,}15\ K$$

$$\Delta G_3^{rota} = 71{,}81\ kJ/mol$$

Figure S2.19: Using the variable temperature NMR and Eyring's equation to find the energy barrier between the major and the minor rotamer in BOD 2 and BOD 3

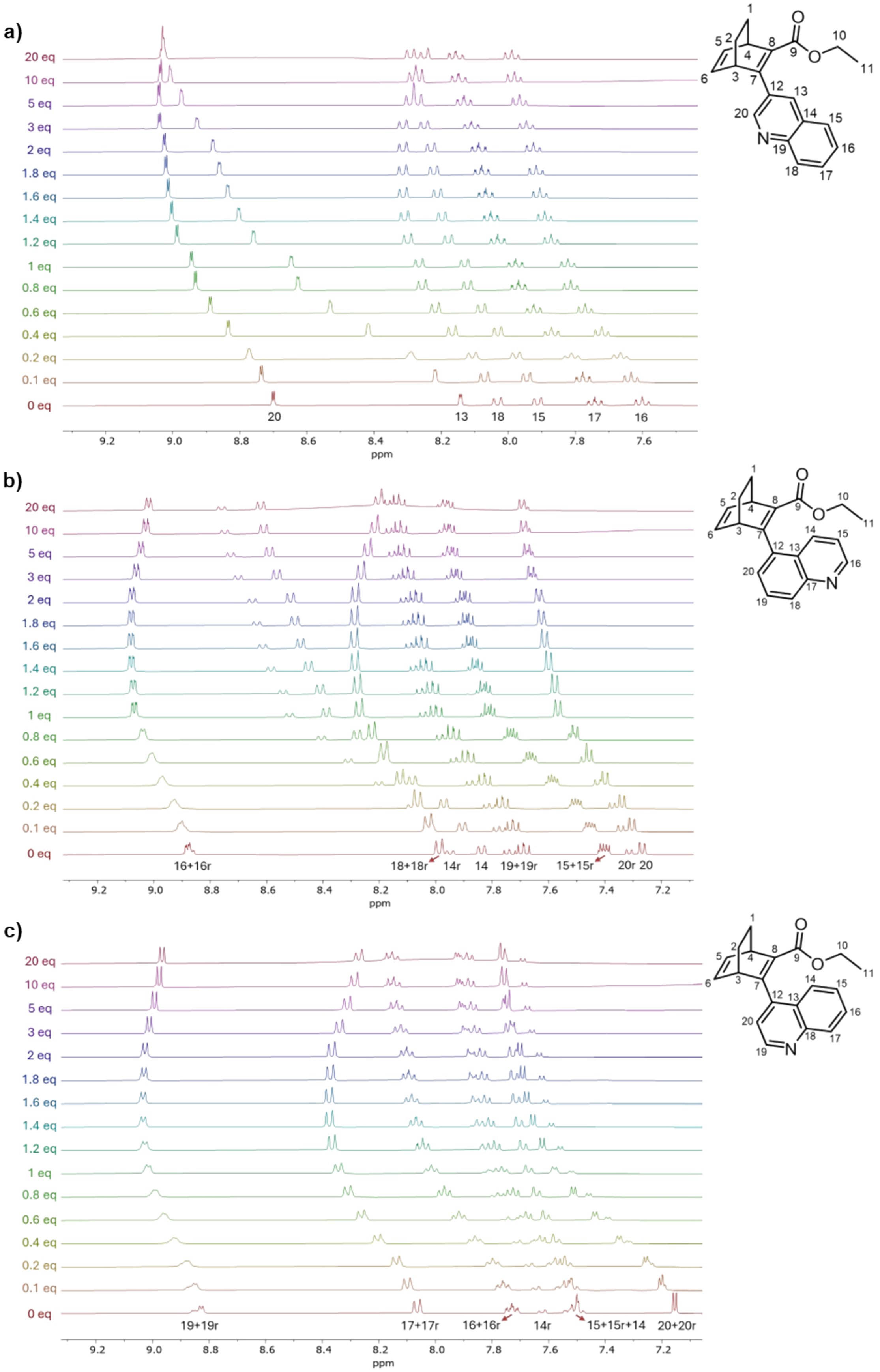

Figure S2.20: ^{1}H-NMR spectra recorded during the gradual addition of acid equivalents (deuterated TFA) in CD_3CN for (a) BOD 1, (b) BOD 2 and (c) BOD 3. The numbers in black correspond to the proton nucleus on the corresponding carbon in the molecule numbering. "r" corresponds to the minor rotamer's signals

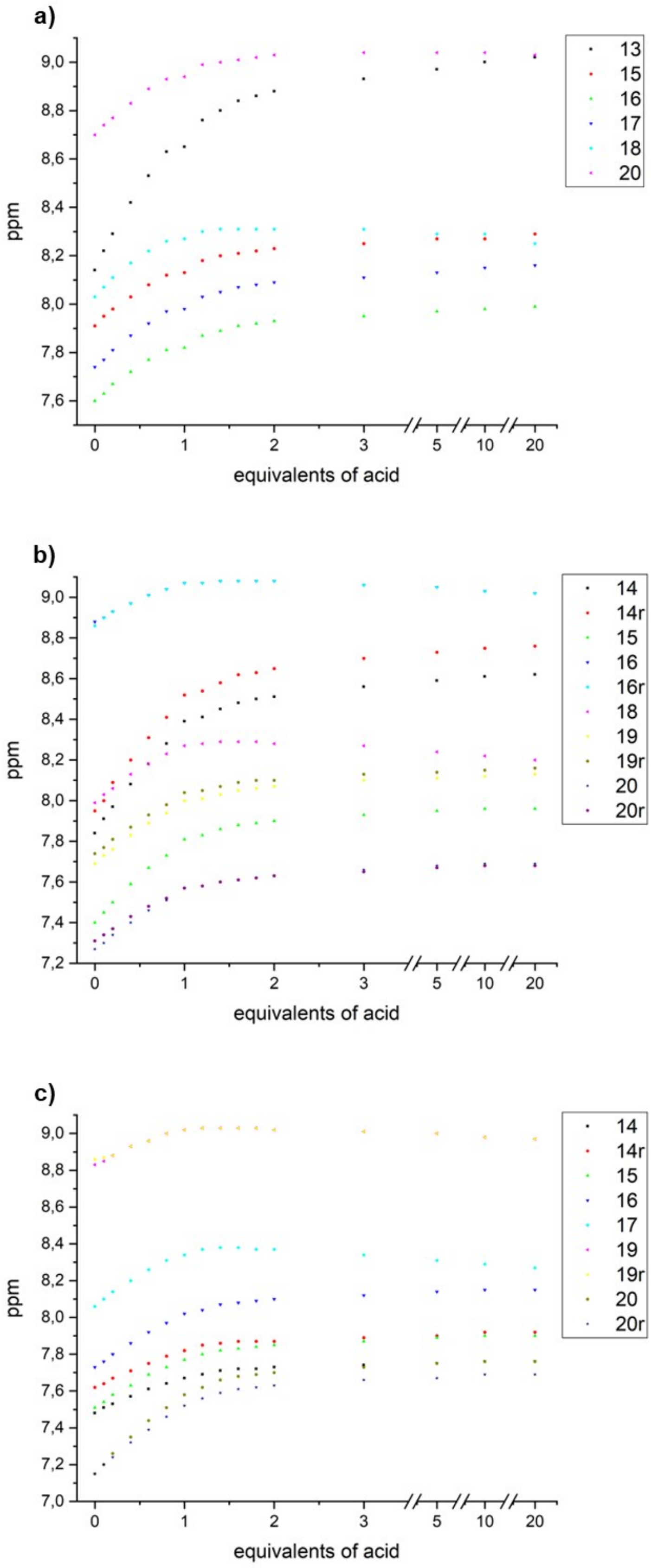

Figure S2.21: Diagram presenting the shift in ppm against the number of acid equivalents added for (a) BOD 1, (b) BOD 2 and (c) BOD 3. "r" corresponds to the minor rotamer's signals

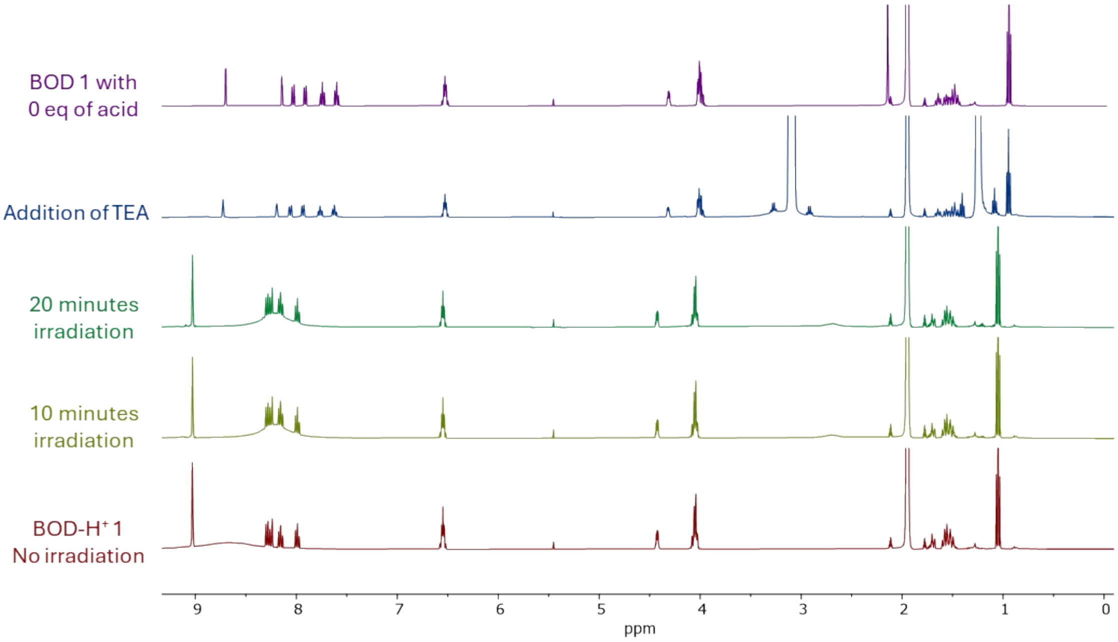

Figure S2.22: ^{1}H-NMR analysis of BOD 1 with 20 equivalents of acid during irradiation with a 365 nm LED with addition of TEA and comparison with BOD 1 without acid

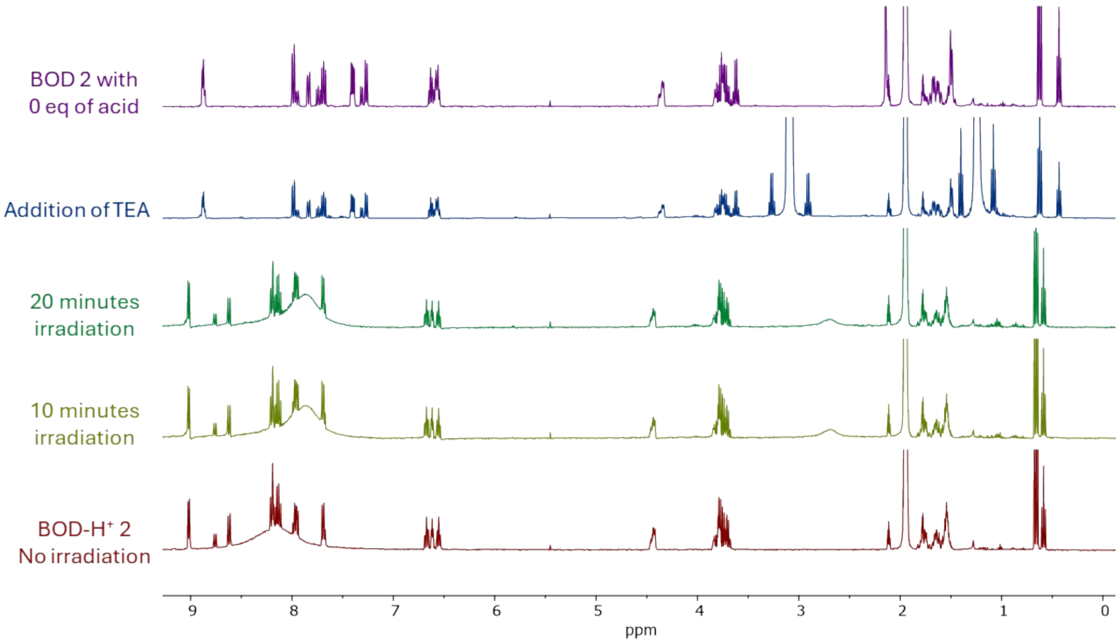

Figure S2.23: ^{1}H-NMR analysis of BOD 2 with 20 equivalents of acid during irradiation with a 365 nm LED with addition of TEA and comparison with BOD 2 without acid

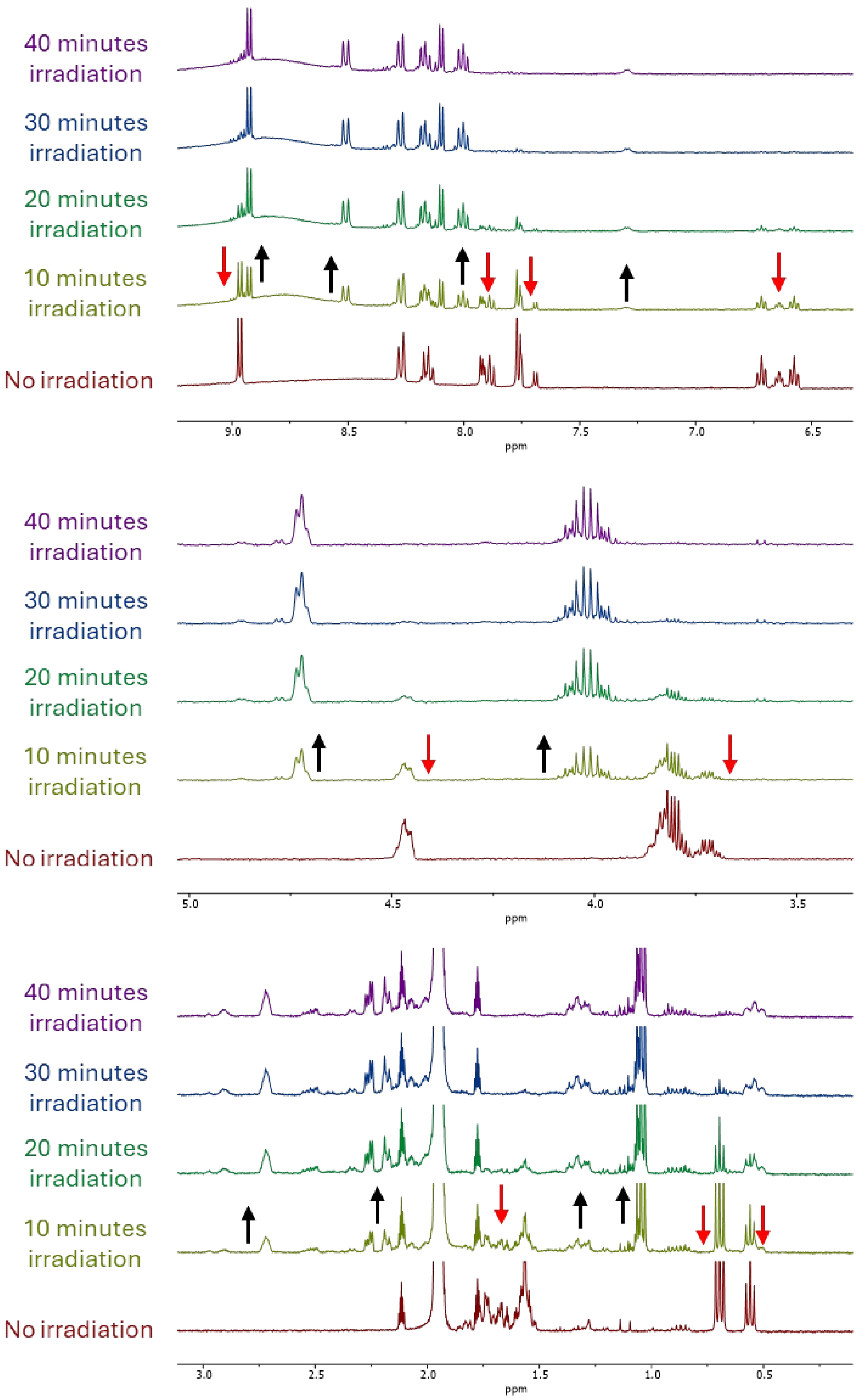

Figure S2.24: ^{1}H-NMR analysis of BOD 3 with 20 equivalents of acid during irradiation with a 365 nm LED

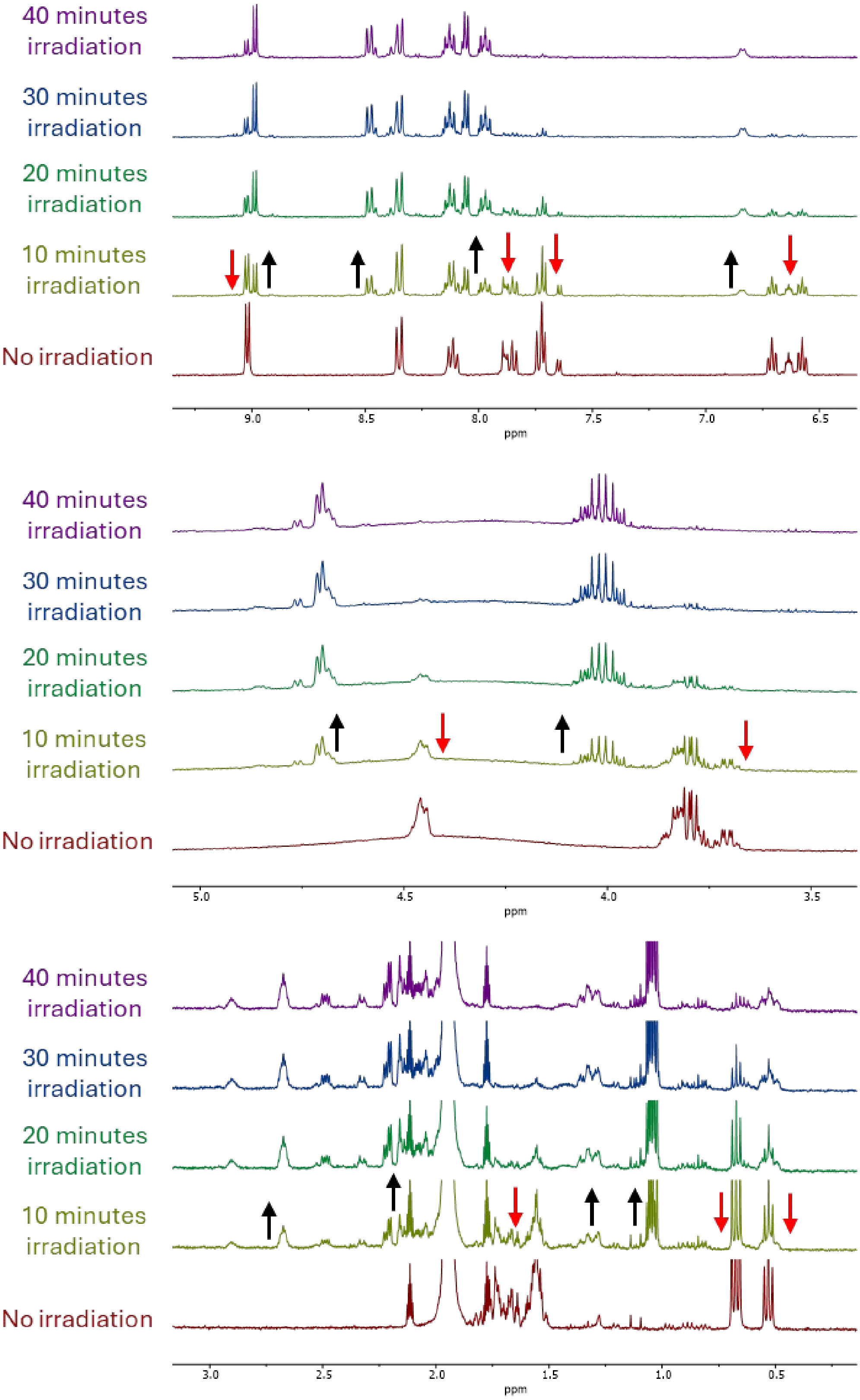

Figure S2.25: ^{1}H-NMR analysis of BOD 3 with 2 equivalents of acid during irradiation with a 365 nm LED

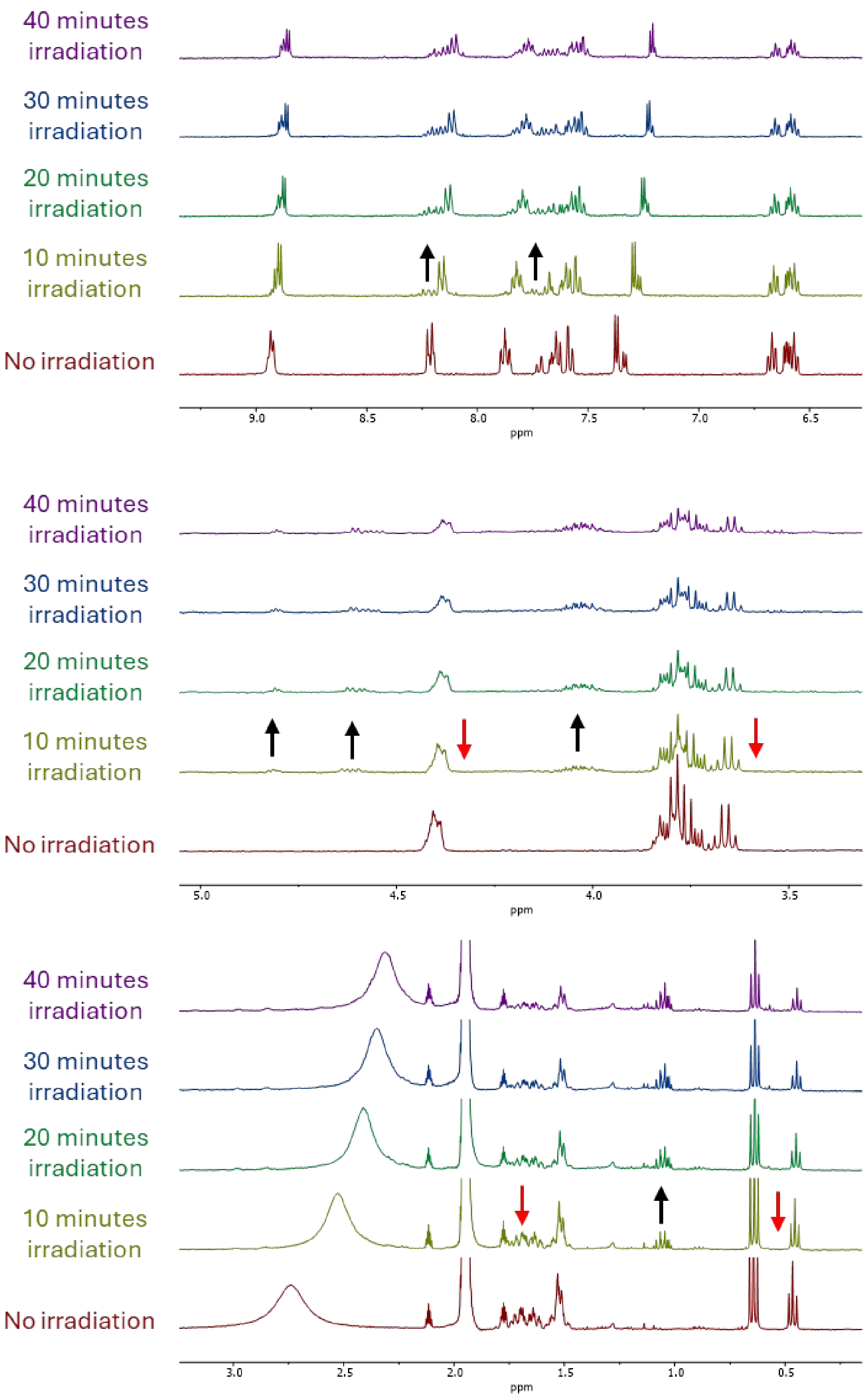

Figure S2.26: ^{1}H-NMR analysis of BOD 3 with 0.5 equivalents of acid during irradiation with a 365 nm LED

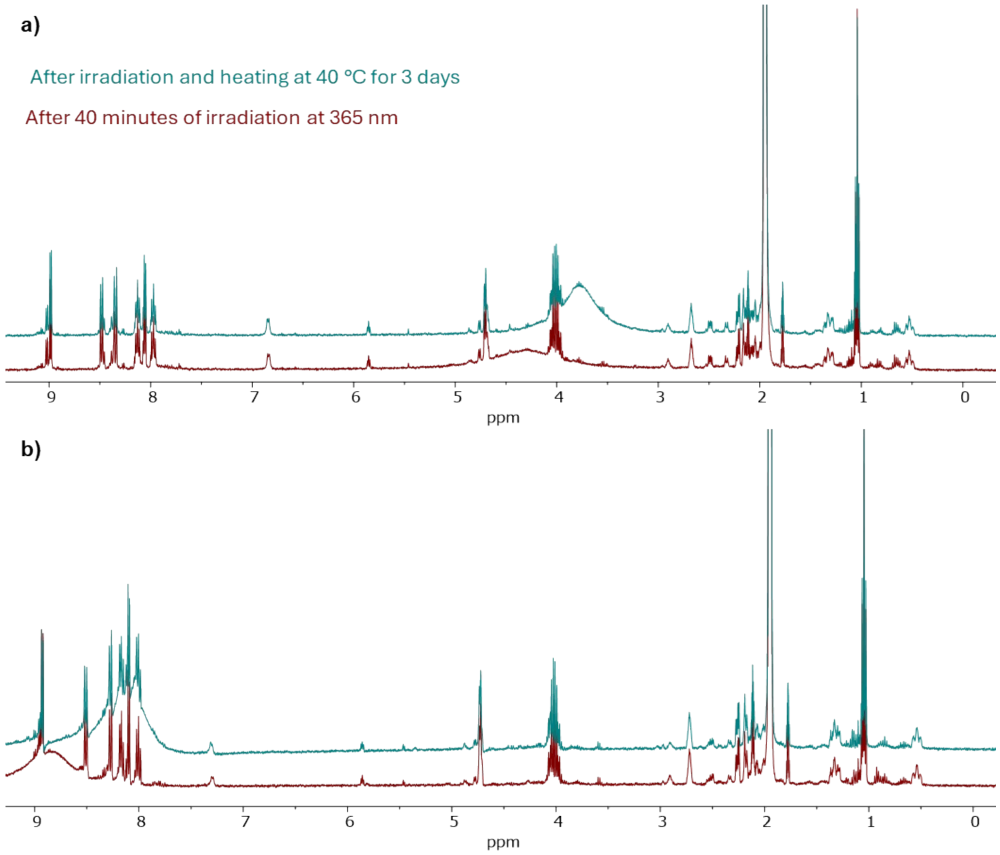

Figure S2.27: Comparison between ^{1}H-NMR spectra in CD3CN of BOD-H+ 3 after 40 minutes of irradiation and after irradiation and heating at 40 °C for three days (a) 2 equivalents of acid, (b) 20 equivalents of acid

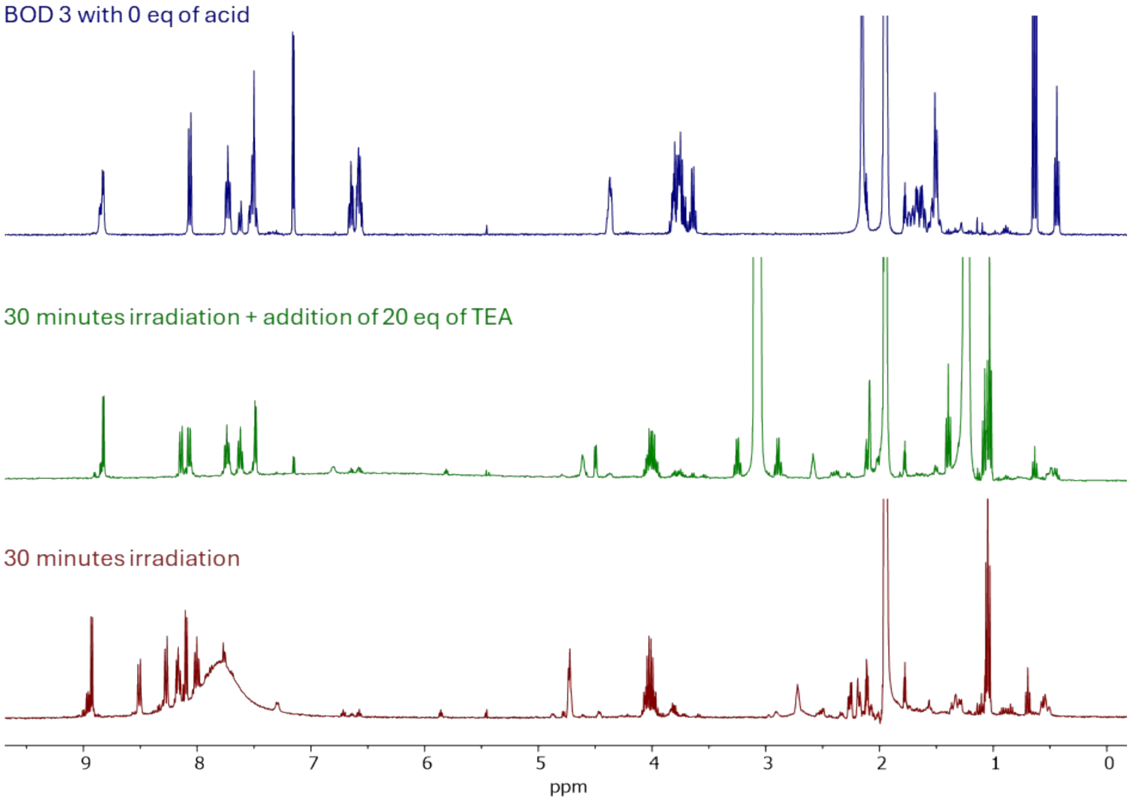

Figure S2.28: NMR Spectra of BOD 3 in CD3CN, after 30 minutes of irradiation, addition of TEA and comparison with BOD 3 without acid

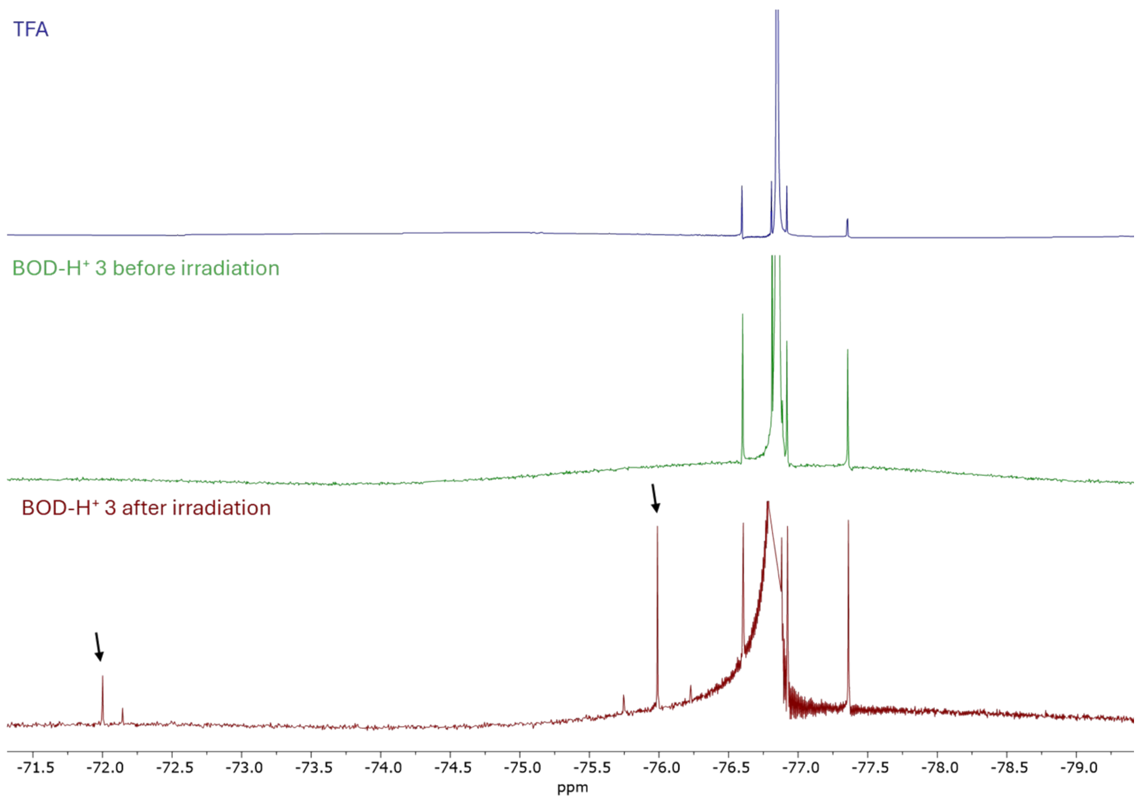

Figure S2.29: ^{19}F-NMR of the TFA used, BOD-H⁺ 3 before and after irradiation in CD_3CN

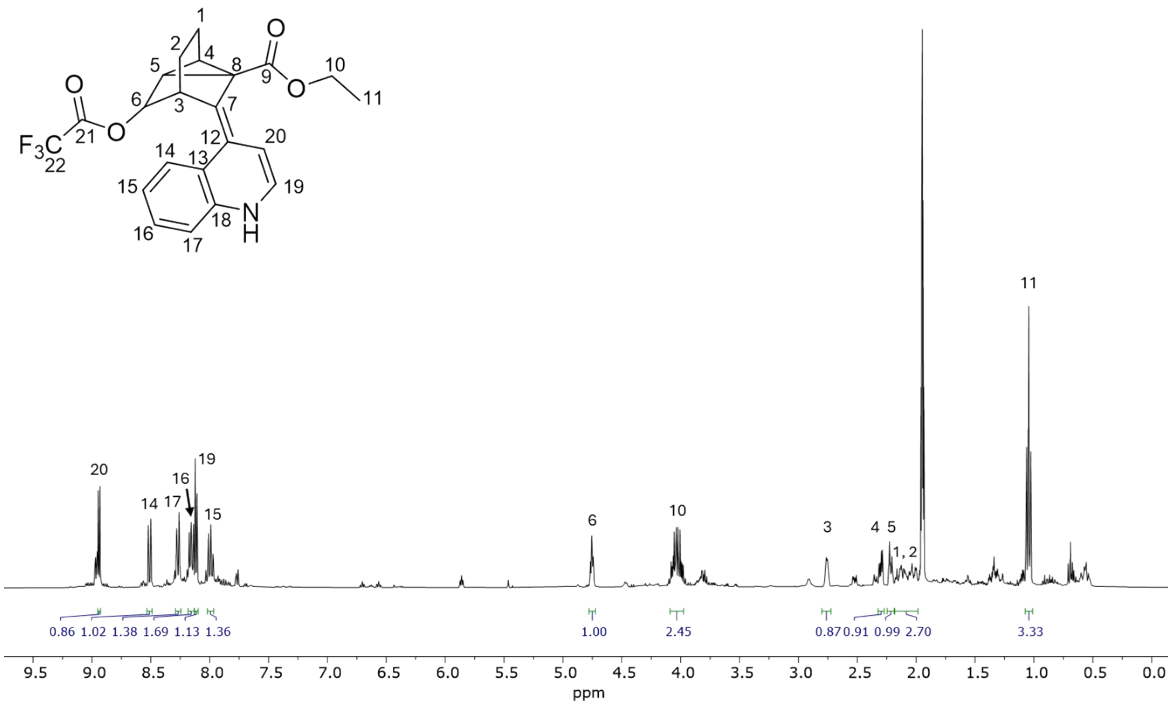

Figure S2.30: ^{1}H-NMR of the degradation product in CD_3CN

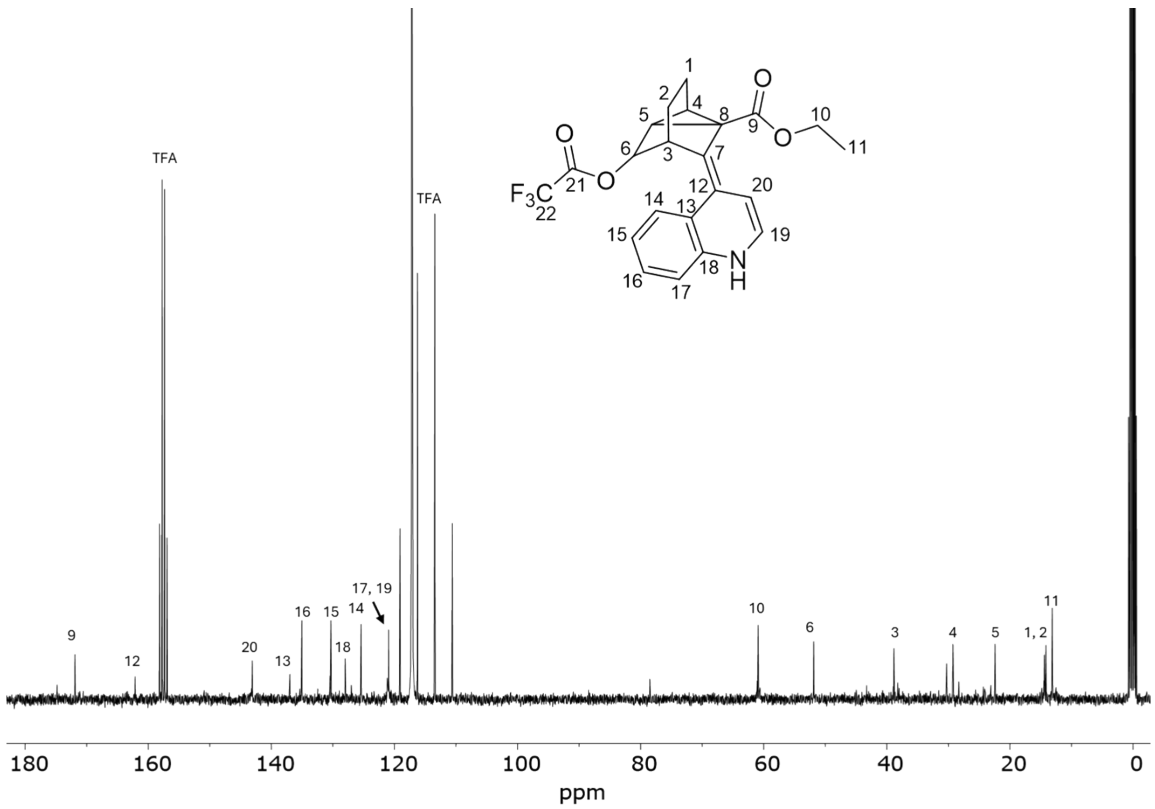

Figure S2.31: ^{13}C-NMR of the degradation product in CD_3CN

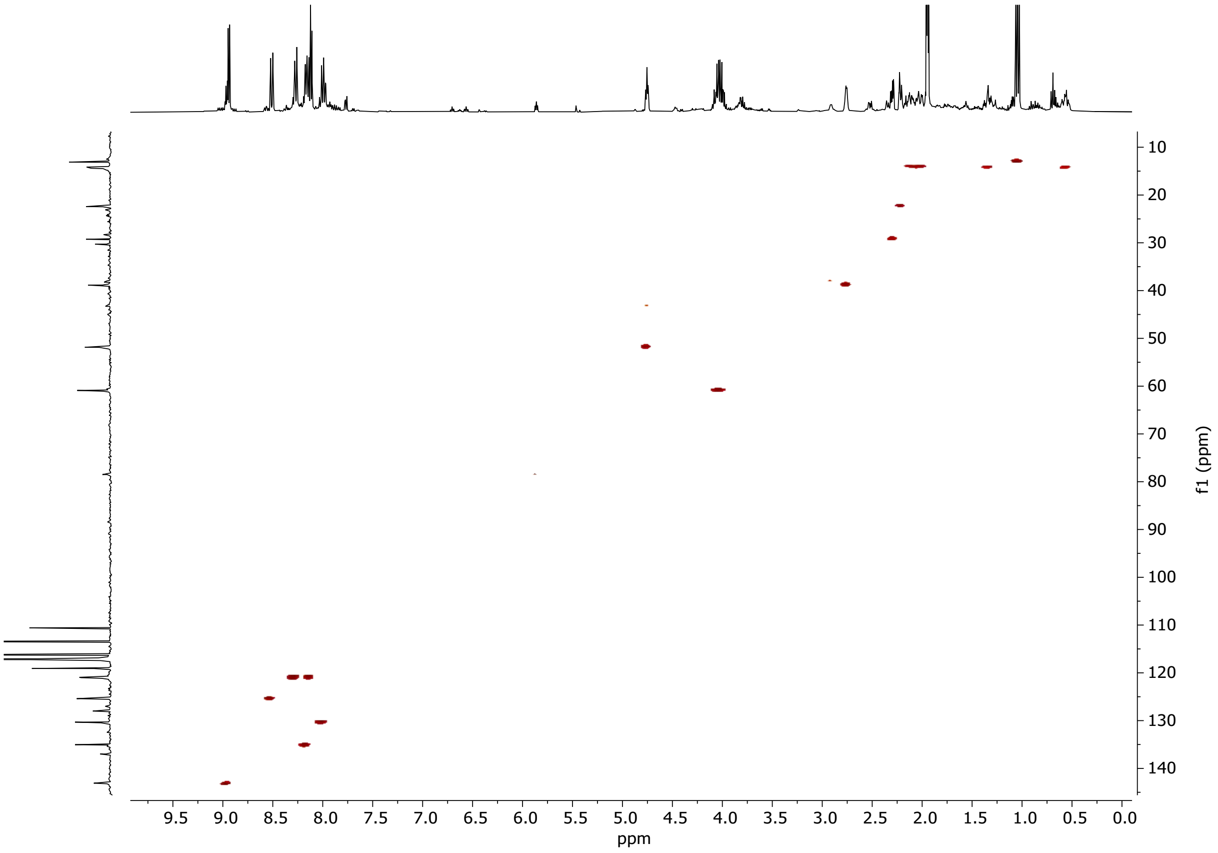

Figure S2.32: HSQC of the degradation product in CD_3CN

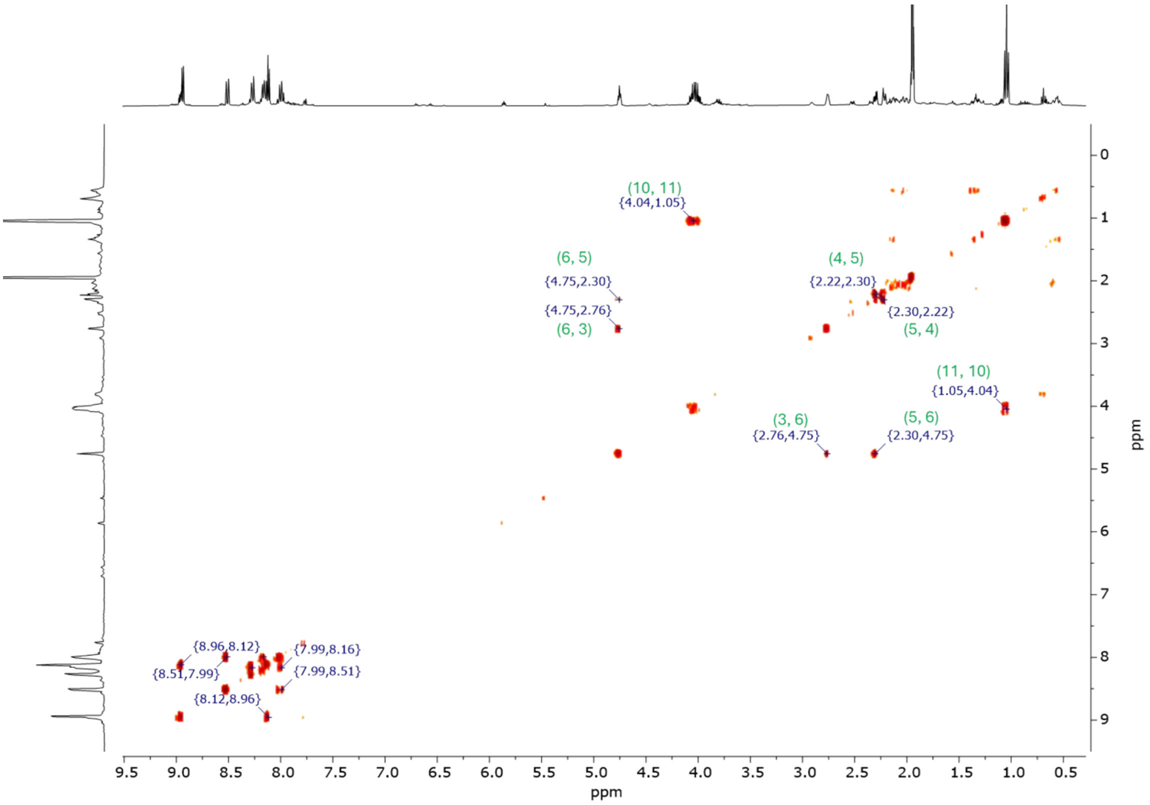

Figure S2.33: COSY of the degradation product in CD_3CN with relevant correlations

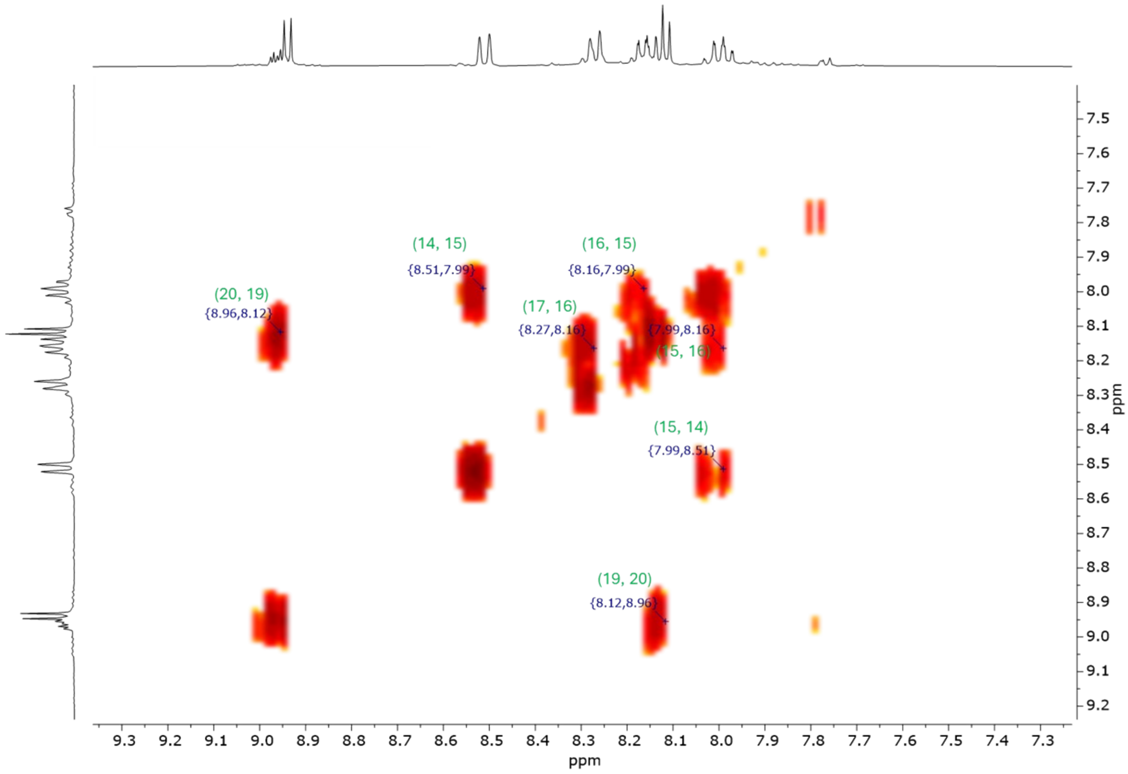

Figure S2.34: COSY of the degradation product in CD3CN with relevant correlations, zoom on the aromatic signals

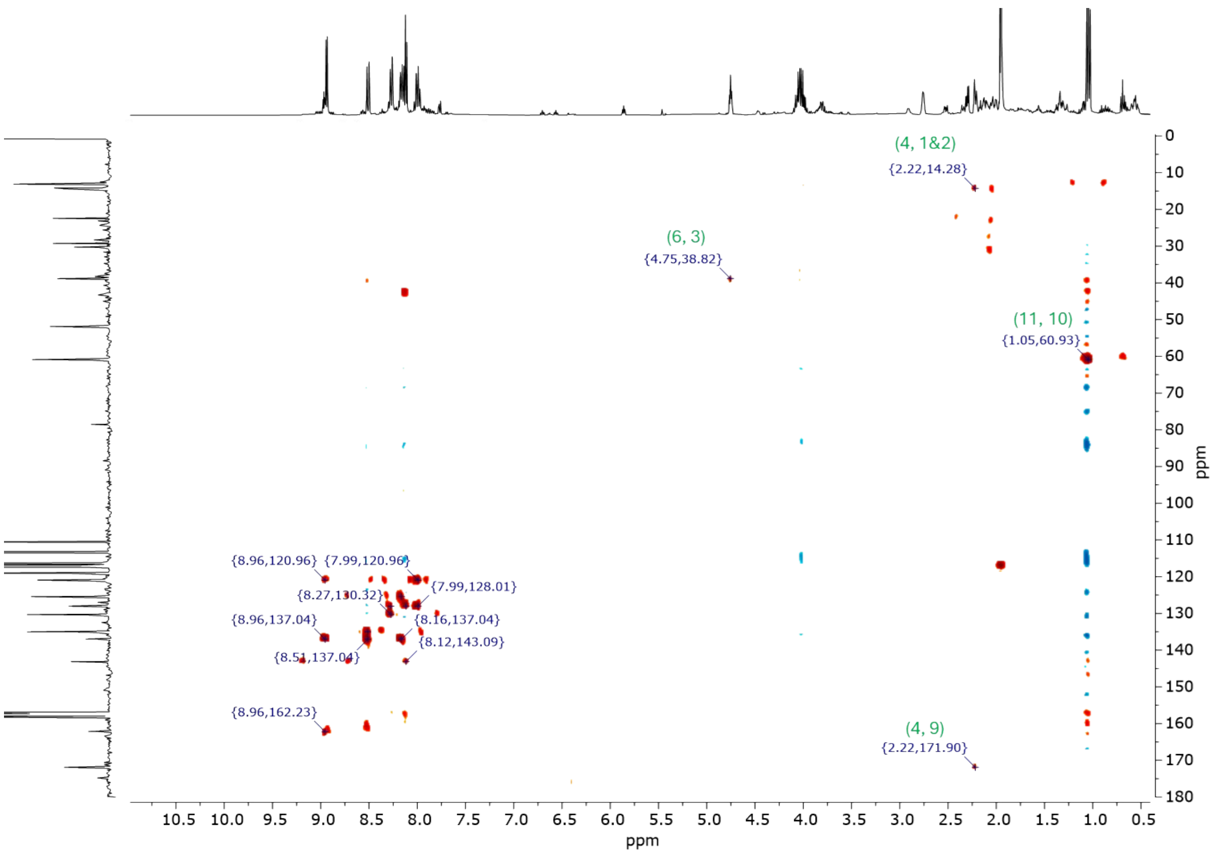

Figure S2.35: HMBC of the degradation product in CD3CN with relevant correlations

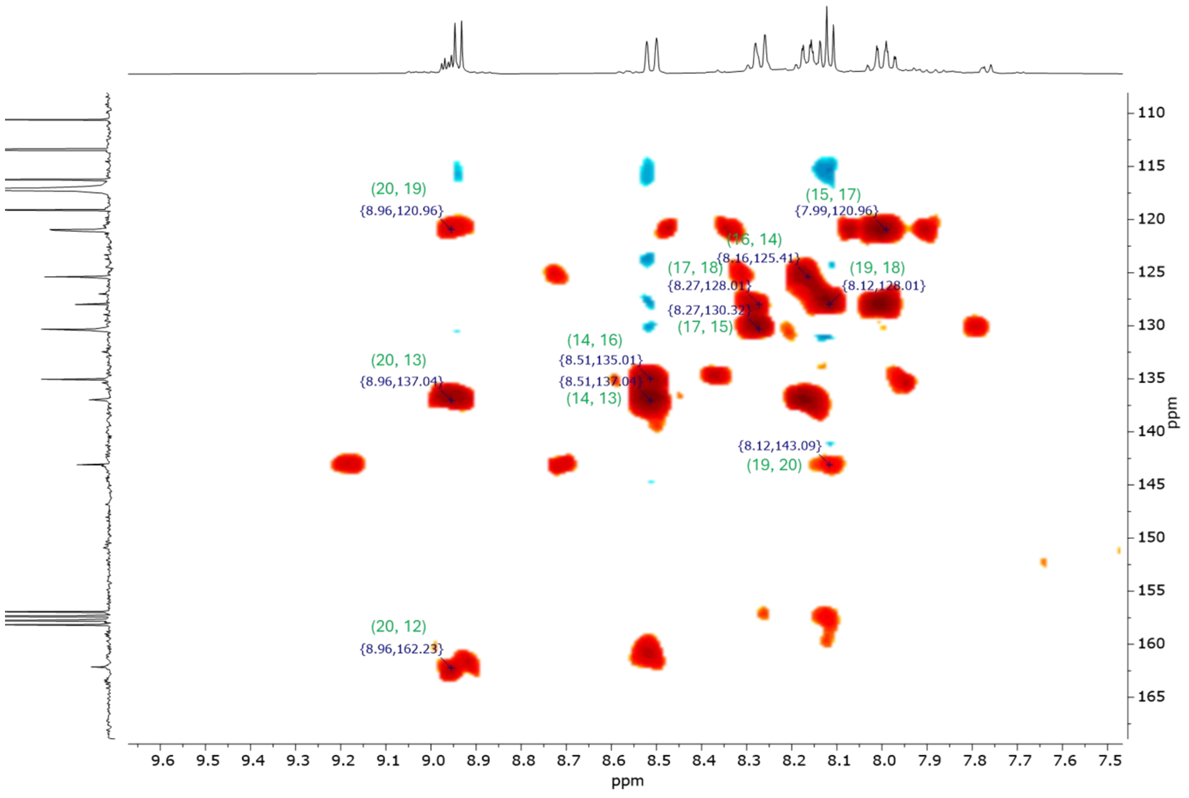

Figure S2.36: HMBC of the degradation product in CD3CN with relevant correlations, zoom on the aromatic signals

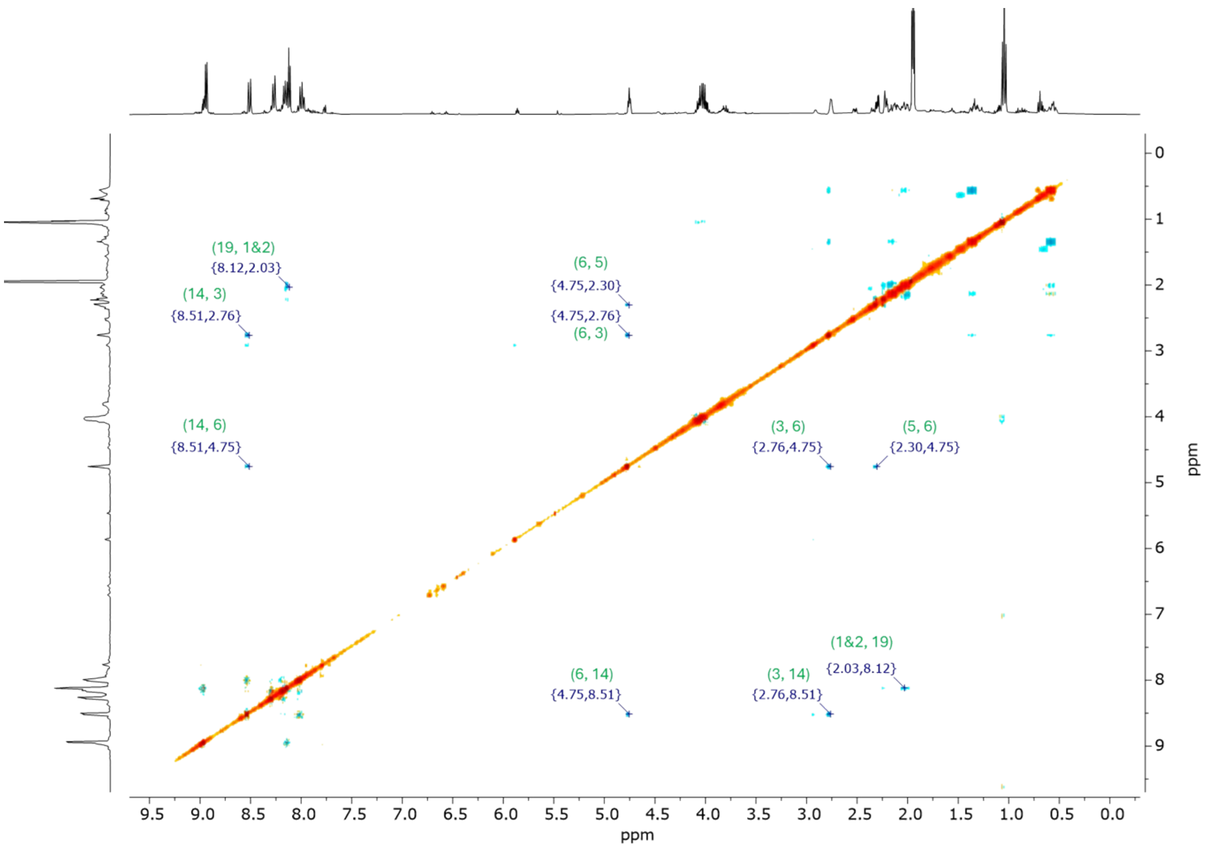

Figure S2.37: NOESY of the degradation product in CD_3CN with relevant correlations

3. Mass spectrometry

All the mass spectrometry analysis were carried out on a Waters Synapt G2 *Si*. The separation by liquid chromatography is achieved using an Acquity UPLC H-Class (Waters, UK), equipped with a non-polar ACQUITY UPLC BEH C18 column (2.1 × 50 mm; 1.7 µm) at 30 °C. The eluant follows a linear gradient starting from 70 % H_2O (with 0.01 % HCOOH)/30 % acetonitrile (ACN) going to 100 % ACN in 10 minutes. HPLC grade solvents were used.

Table S3-1: Exact masses extracted from the UPLC-MS analysis of the irradiation of BOD-H^+ 3 and chemical formula identification

Calculated mass	Measured mass	Chemical formula		Error (ppm)
278.1181	278.1178	$C_{18}H_{16}NO_2$	$M - C_2H_4$	-1.1
306.1494	306.1491	$C_{20}H_{20}NO_2$	M	-1.0
324.1600	324.1599	$C_{20}H_{22}NO_3$	$M + H_2O$	-0.3
368.2059	368.2053	$C_{22}H_{22}D_3N_2O_3$	$M + H_2O + CD_3CN$	1.6
420.1423	420.1425	$C_{22}H_{21}NO_4F_3$	$M + CF_3COOH$	0.5

An ion with *m/z* 445 was also detected during the UPLC-MS but its isotopic model suggests that two boron atoms are present in the structure. Because of its strange chemical formula, this ion is considered as an impurity.

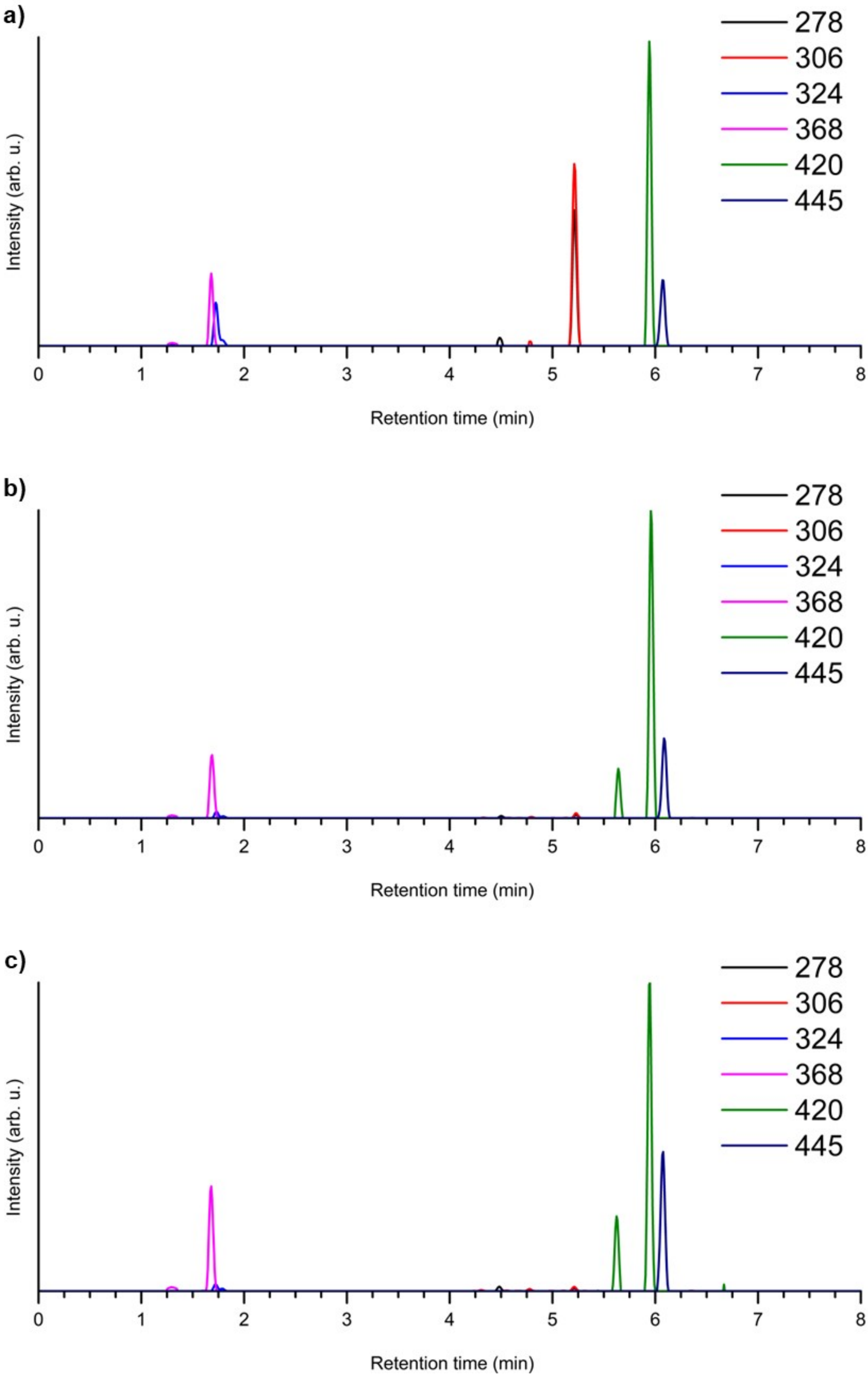

Figure S3.1: Extracted ionic currents from the UPLC-MS analysis of BOD-H^+ 3 after being irradiated in the UV with (a) 0.5 equivalents of acid, (b) 2 equivalents of acid, (c) 20 equivalents of acid

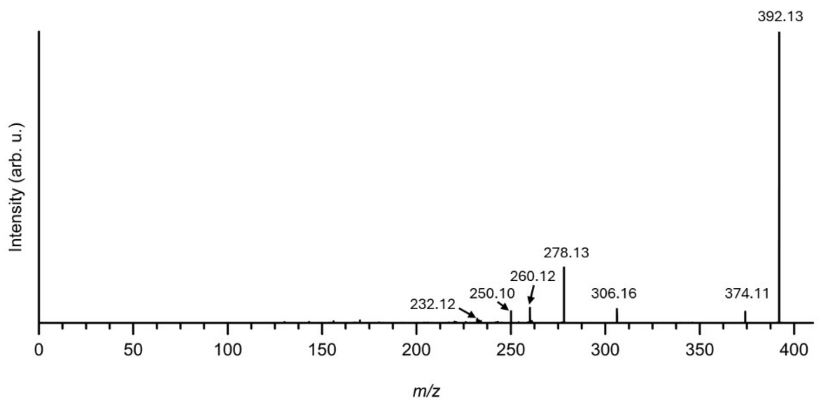

m/z	fragment
392.13	[M+CF3COOH-C2H4+H]$^+$
374.11	[M+CF3COOH-C2H4-H2O+H]$^+$
306.16	[M+H]$^+$
278.13	[M-C2H4+H]$^+$
260.12	[M-C2H4-H2O+H]$^+$
250.10	[M-C2H4-CO2+H]$^+$
232.12	[M-C2H4-HOC2H5+H]$^+$

Figure S3.2: MSMS spectrum of *m/z* 420.16 and the proposed formula of the fragments

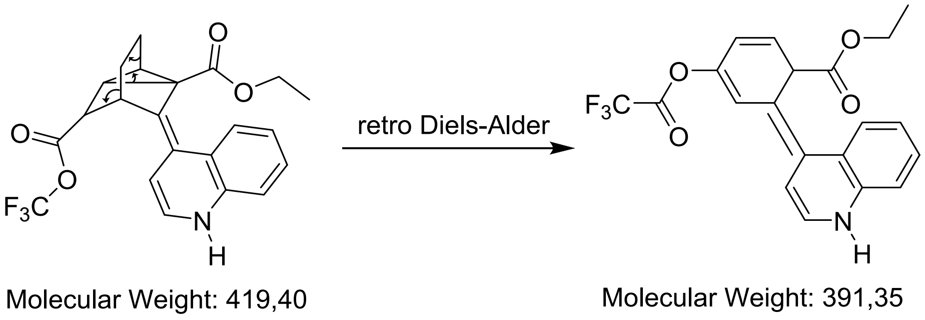

Figure S3.3: Mechanism for the fragmentation of *m/z* 420 into *m/z* 392

4. UV-vis spectroscopy

Molar extinction coefficient

The molar extinction coefficient of the three BODs was determined as the slope of the linear trendline in the graphic of absorbance vs concentration at the maximum of interest for five different concentrations. Here are the trendlines BOD 1, 2, 3 and the shoulders of BOD 2 and 3.

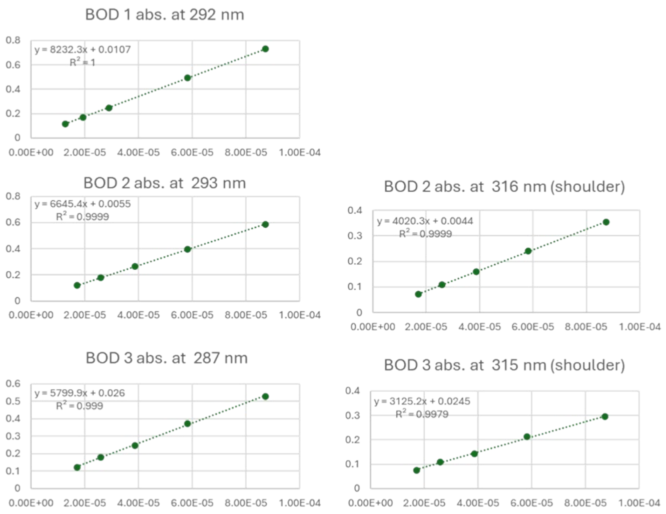

Figure S4.1: Linear fit between Abs. and concentration for the three BODs and the shoulders of BOD 2 and 3

Kinetics of the back conversion

The three BODs were dissolved in acetonitrile and toluene then all solutions were irradiated with a 308 nm LED in a cuvette while the absorption over time was monitored. Diminution of the absorption indicated the switching from BOD to TCO. The end of the switching was shown by a stable absorption over time after which the irradiation was stopped and the absorption over time was still monitored to show the back conversion from TCO to BOD. The measurements were performed at 4 different temperatures for each solution then for each back conversion the rate constant was determined with the Eyring equation which was then plotted in a linear version of the Eyring equation to determine the half-life of the TCOs at 25°C.

The 308 nm LED has a power of 600 mA and the photon fluxes have the following values: 5.01 x 10^{14} s^{-1} in acetonitrile and 5.19 x 10^{14} s^{-1} in toluene.

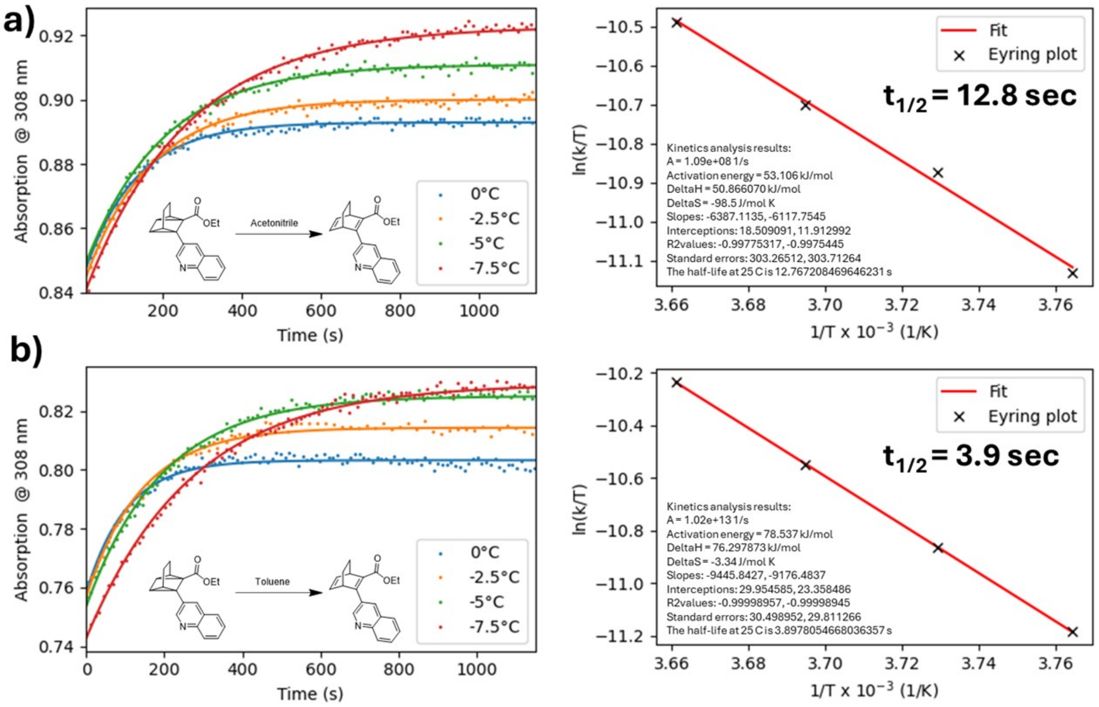

Figure S4.2: Back conversions from TCO 1 to BOD 1 in acetonitrile (a) and toluene (b) and Eyring plots.

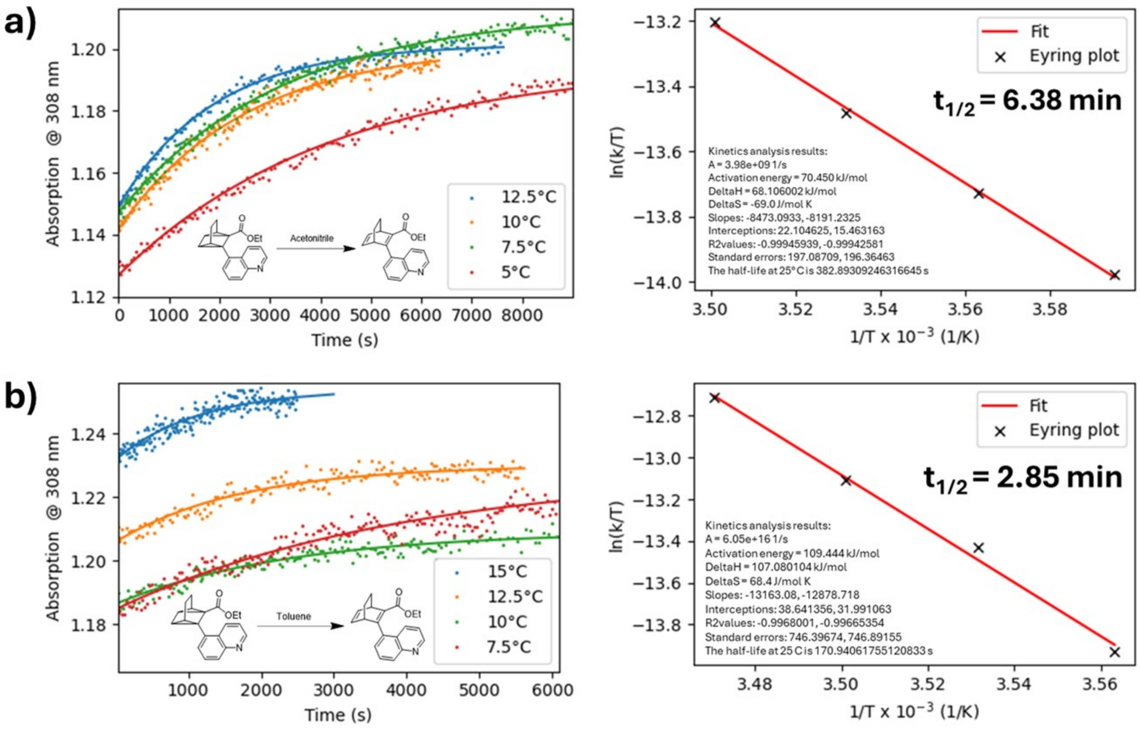

Figure S4.3: Back conversions from TCO 2 to BOD 2 in acetonitrile (a) and toluene (b) and Eyring plots.

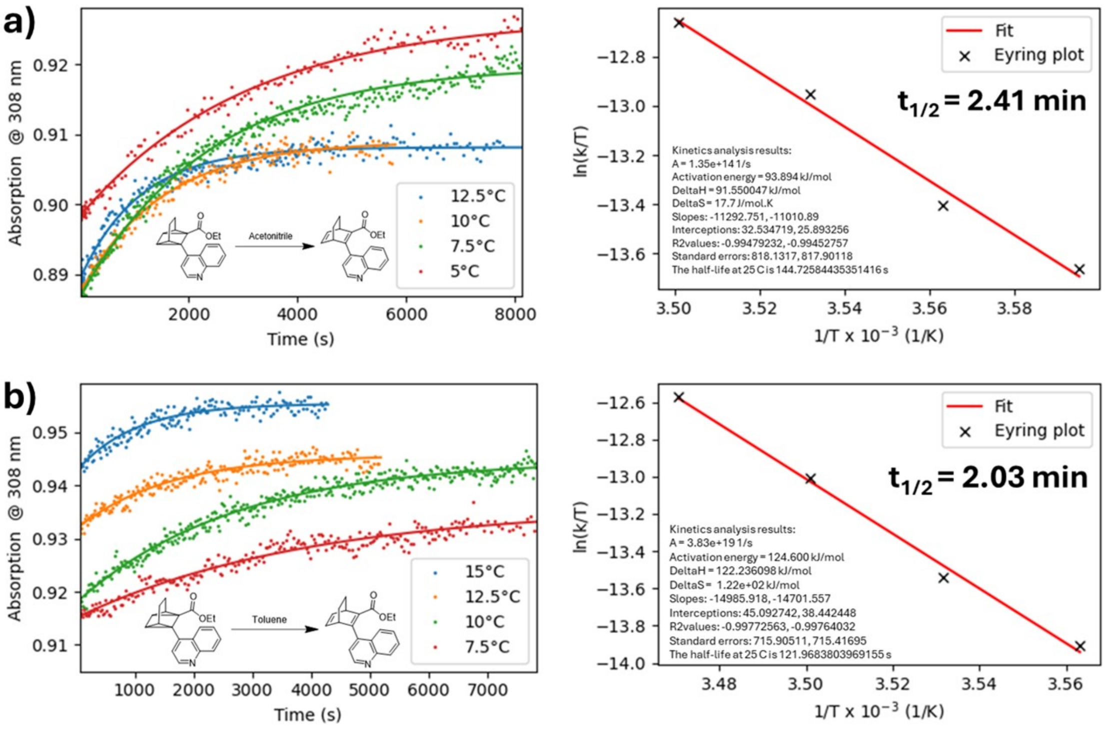

=

Figure S4.4: Back conversions from TCO 3 to BOD 3 in acetonitrile (a) and toluene (b) and Eyring plots.

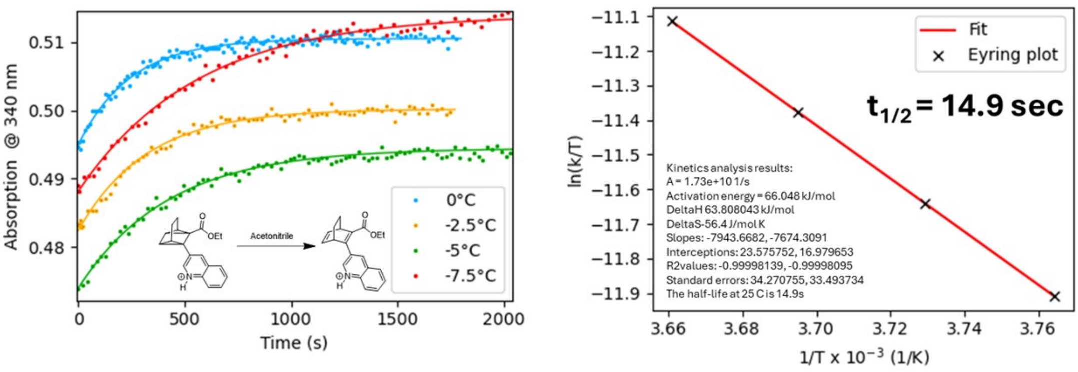

Figure S4.5: Back conversion from TCO-H+ 1 to BOD-H+ 1 in acetonitrile and Eyring plot

Cyclability study

All the cycles were carried out in a normal 3mL cuvette and in the same manner as the kinetic of the back conversion, but the experiment was repeated several times on the same solution to assess the stability of the molecule. Essentially, the absorbance at the beginning and the end of each cycle was plotted. Figure S4.7 shows the temperature effect on the absorbance during the experiment.

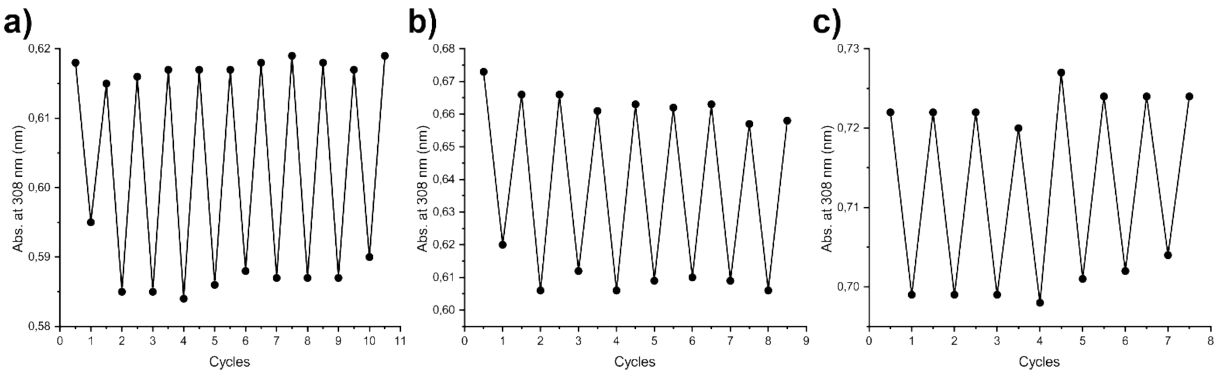

Figure S4.6: Cyclability study of (a) BOD 1, (b) BOD 2 and (c) BOD 3

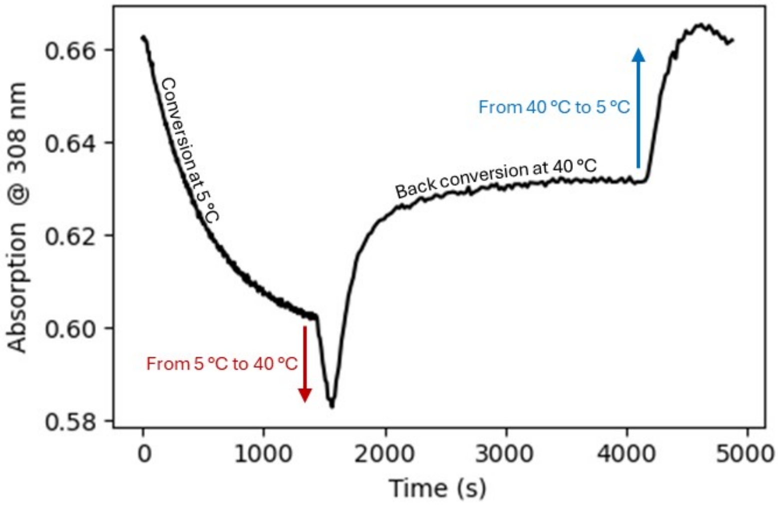

Figure S4.7: Temperature effect encountered during the cyclability study

Quantum yields measurements

For the photoconversion quantum yields of the three BODs, the experiments were carried out in a flow cell with a 80µL volume. For each BOD, the first 30 points of the irradiation were considered to fit the quantum yield. Three experiments were conducted for each BOD to calculate an average quantum yield.

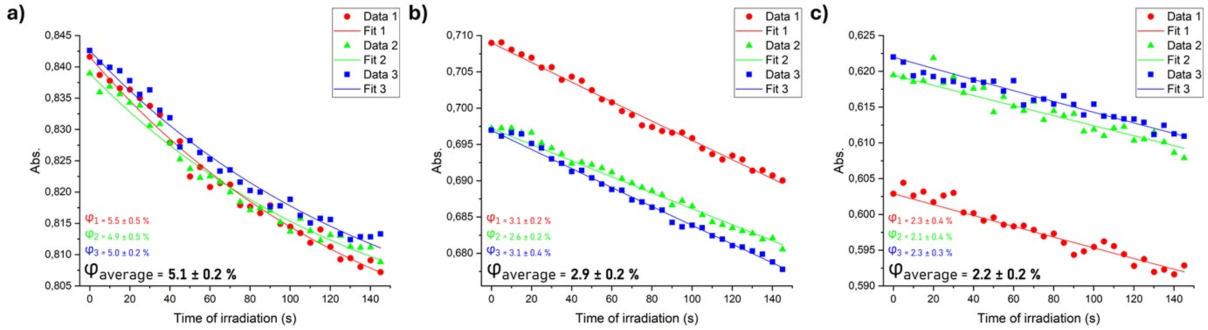

Figure S4.8: Quantum yield measurements in acetonitrile for a) BOD 1, b) BOD 2 , c) BOD 3

Typically, fluorescence quantum yields are calculated using this relative method:

$$\Phi_x = \Phi_r \frac{I_x}{I_r} \frac{A_r}{A_x} \frac{n_x^2}{n_r^2} \frac{t_r}{t_x}$$

where Φ_x and Φ_r are the quantum yields of the sample and reference, respectively, I is the integrated emission intensity, A is the absorbance at the excitation wavelength, n is the refractive index of the solvent, and t is the integration time of the spectrometer. In our case, we used another equation including the slope of a line fitted through the data points of the integrated emission intensity with respect to the absorbance at the excitation wavelength:

$$\phi_f = \phi_f^s \frac{mn^2}{m_s {n_s}^2} \frac{t_{exp}^s}{t_{exp}}$$

where m is the slope of the sample, m_s is the slope of the reference. The data points are collected by measuring the absorbance and emission of the compound at different concentrations. For **BOD-H⁺ 2**, it was done by adding several drops of the compound solution in an acidic acetonitrile solution to keep the equivalence of acid at 20 or above so that the spectra do not change. When the measurements of absorbance and emission were done, more drops were added to change the concentration, and the measurements were conducted again. A similar experiment was conducted for the reference which was coumarin.

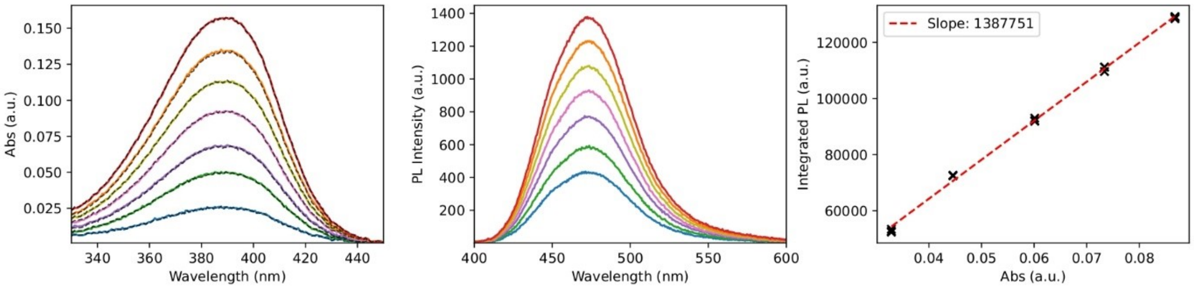

Figure S4.9: Absorbance, photoluminescence (PL) and slope between the integrated PL and absorbance at the excitation wavelength for the reference, coumarin

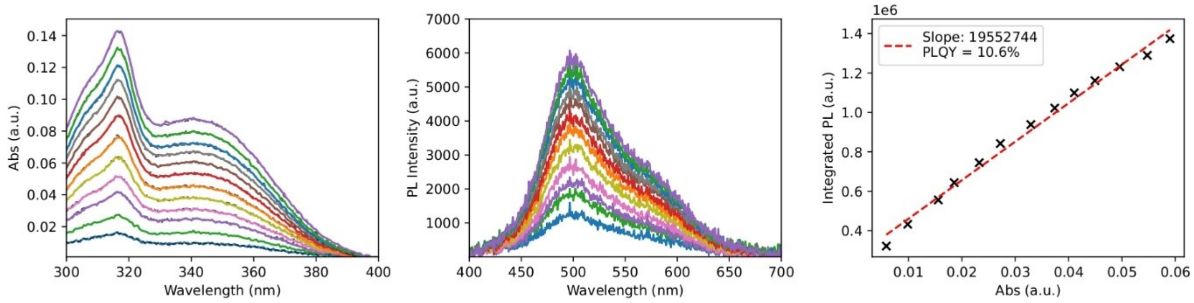

Figure 4.10: Absorbance, photoluminescence (PL) and slope between the integrated PL and absorbance at the excitation wavelength for BOD-H$^+$ 2

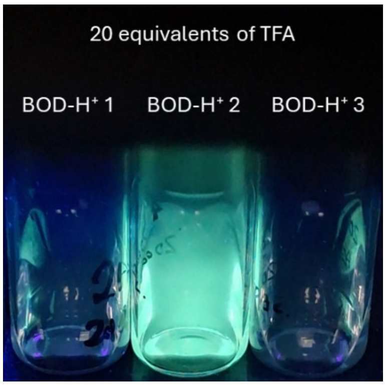

Figure S4.11: Picture of each BOD-H$^+$ with 20 equivalents of TFA under the light of a 365 nm LED

Photoswitching

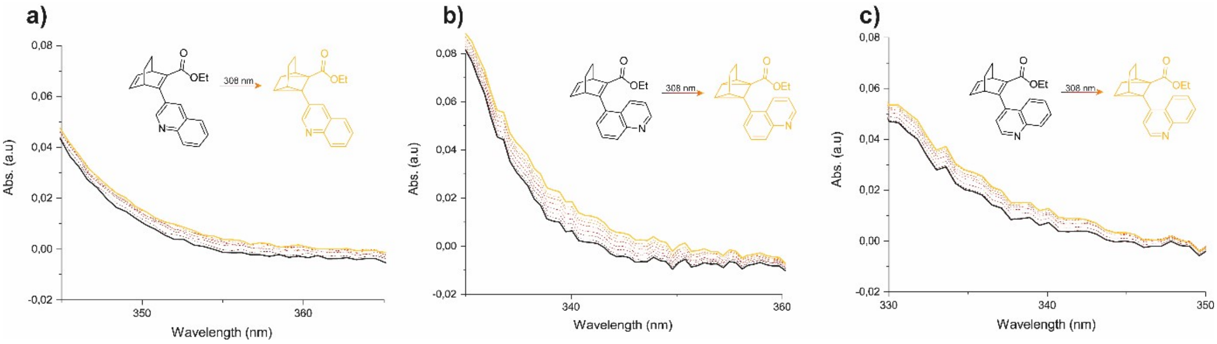

Figure S4.12: Zoom on the UV-vis absorption spectra for the irradiation of (a) BOD 1, (b) BOD 2 and (c) BOD 3 at 0°C in acetonitrile.

5. Theoretical methods

The conformers of each isomer (BOD and TCO) were generated and optimised to find the lowest energy conformer using the Global Optimiser AlgoriThm (GOAT) in Orca 6.0. The lowest energy conformer of each isomer was determined to be the ground state. The energies were calculated with GFN2-xTB in Orca 6.0. The storage energies were determined as the energy difference between the corresponding BOD and TCO. The transition states determined in the scan of the dihedral angle of the quinoline moiety were further optimised with Møller-Plesset second-order perturbation theory (MP2) and the augmented correlation-consistent basis set aug-cc-pVDZ. Furthermore, vertical excitation energies were calculated with time-dependent density functional theory functional B3LYP and the basis set def2-TZVP and the conductor-like polarizable continuum model (CPCM) to simulate solvent effects implicitly. The excitation energies were used to simulate the UV-Vis spectra by broadening the peaks with a Gaussian function.

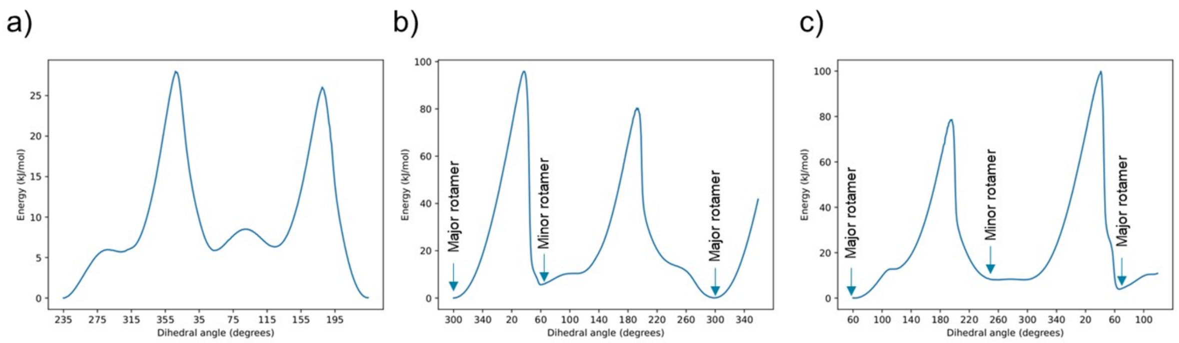

Figure S5.1: Scan of the dihedral angle of the quinoline moiety as a function of the energy of the correspong structures in (a) BOD 1, (b) BOD 2 and (c) BOD 3

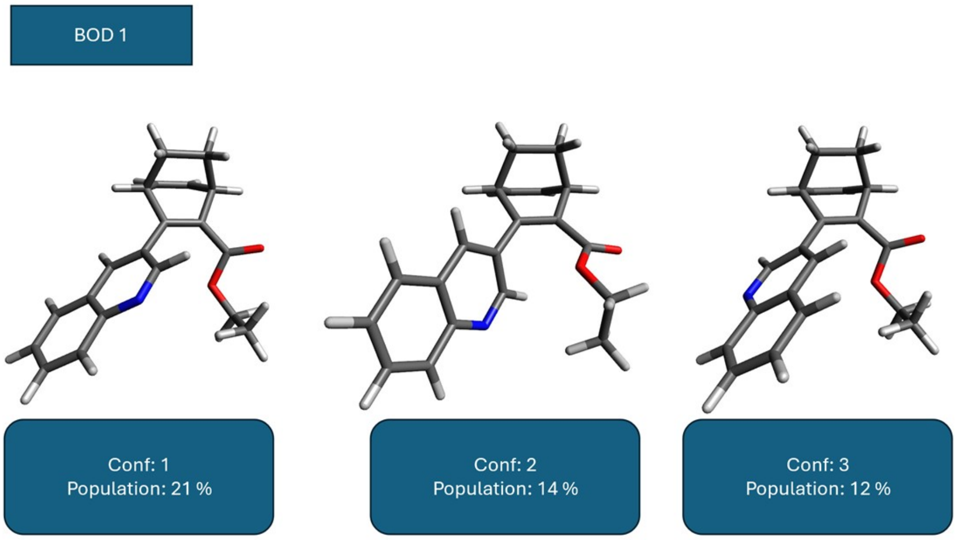

Figure S5.2: 3D structures and population percentage of the 3 most stable conformations of BOD 1

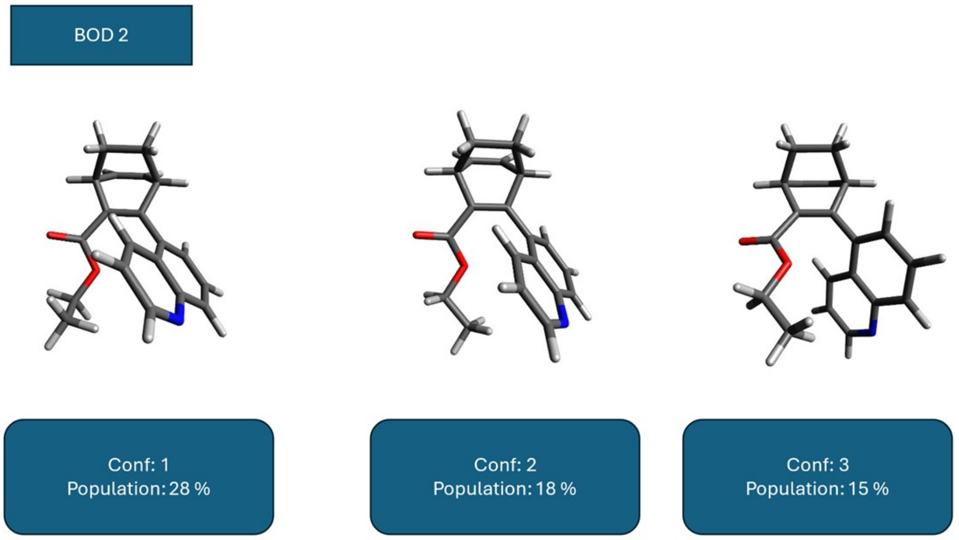

Figure S5.3: 3D structures and population percentage of the 3 most stable conformations of BOD 2

BOD 3

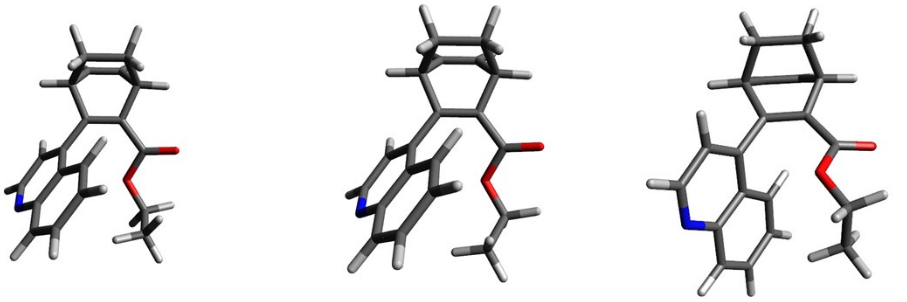

Conf: 1
Population: 27 %

Conf: 2
Population: 22 %

Conf: 3
Population: 21 %

Figure S5.4: 3D structures and population percentage of the 3 most stable conformations of BOD 3

BOD-H⁺ 1

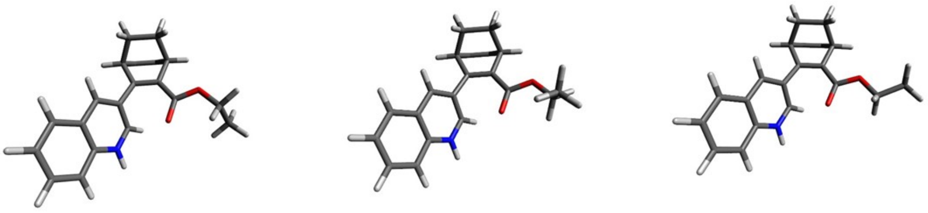

Conf: 1
Population: 14 %

Conf: 2
Population: 13 %

Conf: 3
Population: 11 %

Figure S5.5: 3D structures and population percentage of the 3 most stable conformations of BOD-H^+ 1

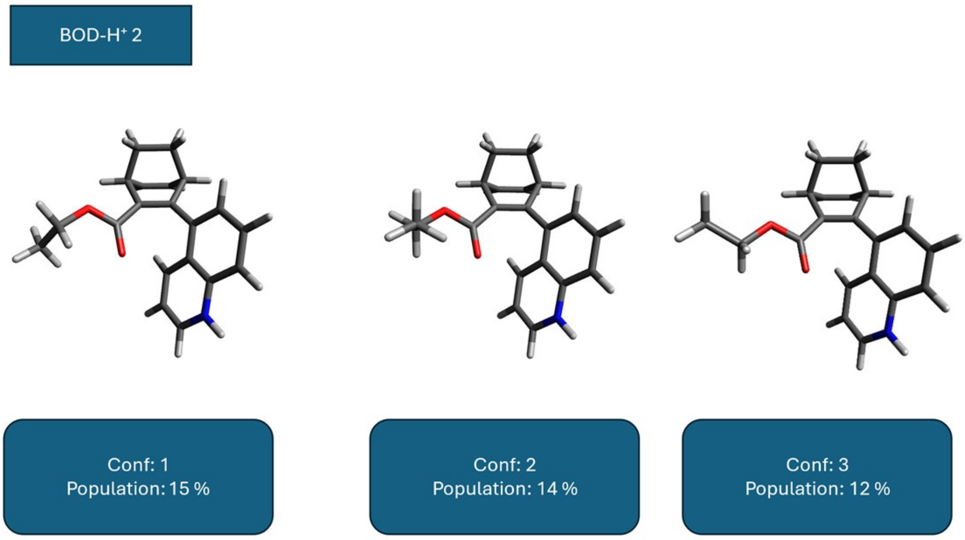

Figure S5.6: 3D structures and population percentage of the 3 most stable conformations of BOD-H^+ 2

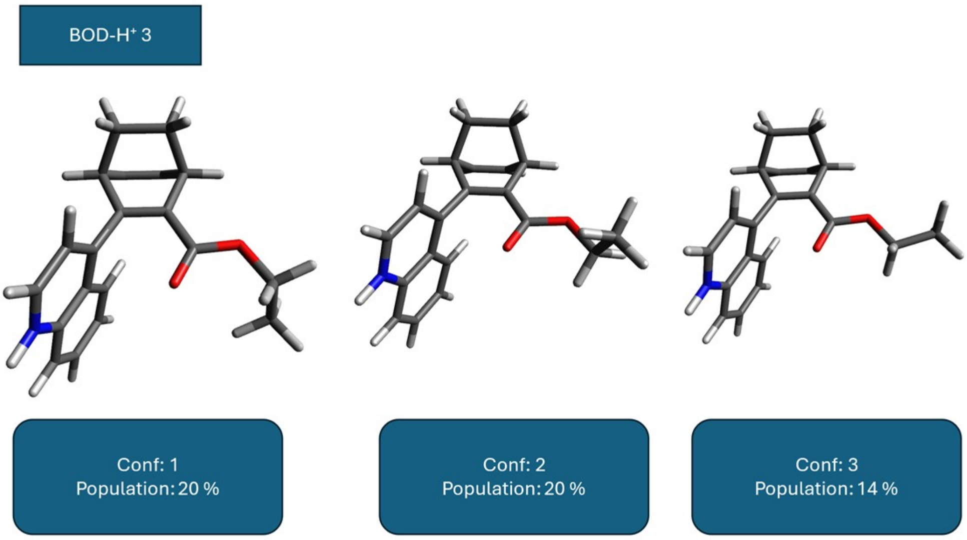

Figure S5.7: 3D structures and population percentage of the 3 most stable conformations of BOD-H^+ 3

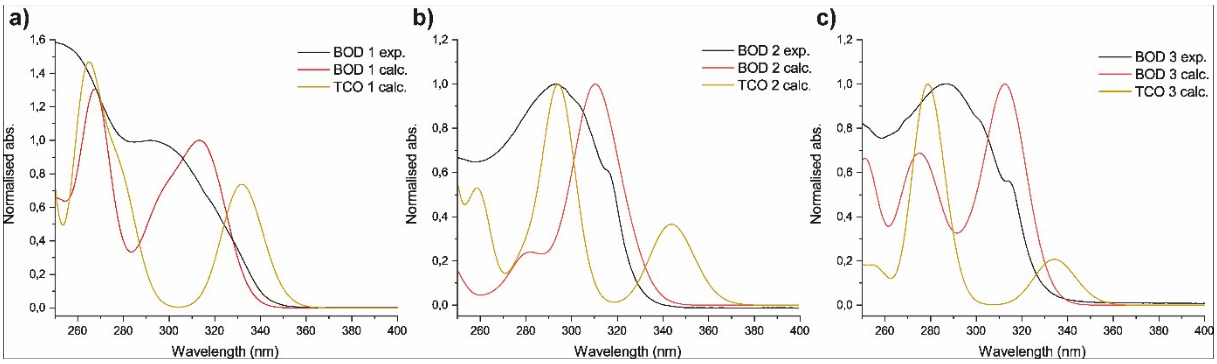

Figure S5.8: Comparison between the calculated and experimental absorbance spectra in acetonitrile of a) BOD 1 and TCO 1, b) BOD 2 and TCO 2, c) BOD 3 and TCO 3
